# The *Fusarium oxysporum gnt2*, Encoding a Putative *N*-Acetylglucosamine Transferase, Is Involved in Cell Wall Architecture and Virulence

**DOI:** 10.1371/journal.pone.0084690

**Published:** 2013-12-27

**Authors:** Loida López-Fernández, Carmen Ruiz-Roldán, Yolanda Pareja-Jaime, Alicia Prieto, Husam Khraiwesh, M. Isabel G. Roncero

**Affiliations:** 1 Departamento de Genética, Universidad de Córdoba, Córdoba, Spain; 2 Campus de Excelencia Agroalimentario (ceiA3), Córdoba, Spain; 3 Centro de Investigaciones Biológicas-CSIC, Madrid, Spain; 4 Departamento de Biología Celular, Fisiología e Inmunología, Universidad de Córdoba, Córdoba, Spain; University of Helsinki, Finland

## Abstract

With the aim to decipher the molecular dialogue and cross talk between *Fusarium oxysporum* f.sp. *lycopersci* and its host during infection and to understand the molecular bases that govern fungal pathogenicity, we analysed genes presumably encoding *N-*acetylglucosaminyl transferases, involved in glycosylation of glycoproteins, glycolipids, proteoglycans or small molecule acceptors in other microorganisms. *In silico* analysis revealed the existence of seven putative *N-*glycosyl transferase encoding genes (named *gnt*) in *F. oxysporum* f.sp. *lycopersici* genome. *gnt2* deletion mutants showed a dramatic reduction in virulence on both plant and animal hosts. Δ*gnt2* mutants had αalterations in cell wall properties related to terminal αor β-linked *N-*acetyl glucosamine. Mutant conidia and germlings also showed differences in structure and physicochemical surface properties. Conidial and hyphal aggregation differed between the mutant and wild type strains, in a pH independent manner. Transmission electron micrographs of germlings showed strong cell-to-cell adherence and the presence of an extracellular chemical matrix. Δ*gnt2* cell walls presented a significant reduction in *N*-linked oligosaccharides, suggesting the involvement of Gnt2 in *N*-glycosylation of cell wall proteins. Gnt2 was localized in Golgi-like sub-cellular compartments as determined by fluorescence microscopy of GFP::Gnt2 fusion protein after treatment with the antibiotic brefeldin A or by staining with fluorescent sphingolipid BODIPY-TR ceramide. Furthermore, density gradient ultracentrifugation allowed co-localization of GFP::Gnt2 fusion protein and Vps10p in subcellular fractions enriched in Golgi specific enzymatic activities. Our results suggest that *N-*acetylglucosaminyl transferases are key components for cell wall structure and influence interactions of *F. oxysporum* with both plant and animal hosts during pathogenicity.

## Introduction

The amino sugar *N*-acetylglucosamine (GlcNAc) plays important roles in a wide range of organisms from bacteria to humans. One major role for GlcNAc is shaping the structure of the extracellular cell surface. GlcNAc is converted to UDP-GlcNAc, which is a substrate for the transfer of the GlcNAc moiety to macromolecules. In fungi, UDP-GlcNAc is the substrate for chitin synthases that form the cell wall chitin, a β-(1→4) GlcNAc polymer. 

In the fungal plant pathogen *Fusarium oxysporum*, integrity of the cell wall structure has been associated with plant interaction [1]. Chitin plays an important role in the pathotypic behaviour toward tomato plants (*Lycopersicon esculentum*). Deletion of the Chs V class V chitin synthase demonstrated that this enzyme plays different roles in fungal pathogenesis on plants [2] and mammalian systems [3]. Evidence has been obtained showing that perturbation of fungal cell wall biosynthesis causes avirulence by elicitation of the induced plant defence-response leading to the restriction of fungal infection [4].

UDP-GlcNAc is also used in eukaryotes to initiate *N-*linked glycosylation and for the synthesis of glycosyl-phosphatidylinositol (GPI) anchors on membrane proteins. In addition to its structural roles, GlcNAc is an important signalling molecule in bacteria, fungi and animal cells. The glycosylation reaction is catalysed by the action of glycosyl transferases, which transfer different mono-saccharides from nucleotide activated sugar donors in α-conformation to various glycans on glycoproteins, glycolipids, proteoglycans or small molecule acceptors [5]. Oligosaccharide structures attached to proteins are conserved in eukaryotes, being one of the most abundant post-translational modification reactions [6]. Glycosylation plays numerous roles in protein folding and conformation, targeting, recognition, and other biological functions. Changes in glycan structures are associated with many physiological and pathological events, including cell adhesion, migration, cell growth, cell differentiation, and tumour invasion [7,8]. Oligosaccharides of glycoproteins are classified as *N-*glycans and *O-*glycans [9]. *N-*linked protein glycosylation, present in all domains of life, has two characteristics in common: the oligosaccharide is preassembled on a lipid carrier (dolichyl pyrophosphate), and then transferred in bloc to an asparagine residue within the consensus sequence Asn- X- Ser/Thr of the protein, as opposed to *O-*glycans which are attached to a subset of Ser and Thr [10,11]. Once the *N*-glycoproteins have been correctly folded and passed the endoplasmic reticulum (ER) quality control mechanism, they are transported to the Golgi where they are further modified [12–14]. Among the enzymes involved in the glycosylation process, the *N-*acetylglucosaminyl transferases (GnTs) transfer *N-*acetyl glucosamine residues from UDP-GlcNAc to the specific acceptor protein*-*linked structures, converting them into hybrid or complex glycan types.

Protein glycosylation pathways of 12 filamentous fungal species were investigated using a systems biology approach and developing a composite representation [15]. The *N-*glycosylation pathway in the cytoplasm and ER was evolutionarily conserved across the species studied, and highly specialized *N-*glycan structures with galactofuranose residues, phosphodiesters, and other insufficiently trimmed structures were identified.

In the basidiomycete *Coprinopsis cinerea, N-*glycans of cell wall proteins from the fruiting body have been characterized [16]. The authors identified a novel oligosaccharide structure with at least five mannoses and a bisecting α, 1→4 *N-*acetylglucosamine linked to the -mannose of the *N-*glycan core, resembling a bisecting-hybrid-type glycan as in higher eukaryotes. The transferase responsible for this modification, CcGnt1, was described as a retaining glycosyltransferase from family 8 (GT8), as classified in the CaZY database [17], and predicted to be a type II membrane protein. 

In the model fungus *S. cerevisiae*, protein glycosylation has been extensively studied for decades, revealing much of the enzymology of both Golgi and ER glycosylation pathways. Several authors examined the oligosaccharides attached to endogenous proteins, including invertase, exoglucanase and carboxypeptidase Y [18–20]. These studies indicated that the structure of yeast *N-*linked glycans is based solely on mannose and phosphomannose, without evidence for the addition of further *N-*acetylhexosamine residues beyond the two GlcNAc residues of the core structure. Nevertheless, an ortholog of the *GNT1* gene (*YOR320c*) encoding an open reading frame related to known *N-*acetylglucosaminyl transferases has been identified. Deletion of this ORF resulted in loss of the extra mass on the *N-*linked glycans and of lectin binding [21]. The phenotype of yeast mutants lacking *GNT1* provided few clues for its likely function. The *gnt1* mutants showed no change in sensitivity to caffeine, calcofluor white, or hygromycin, all of which have increased toxicity toward strains with cell wall defects [22], and there was no change in the mobility of invertase, or increased secretion of the ER resident protein Kar2p. 

In the human pathogens *Candida albicans*, *Cryptococcus neoformans* and *Aspergillus fumigatus*, glycosylation is essential for virulence [23–31]. Although the role of glycosylation is poorly understood in plant pathogens, it was recently shown to be crucial for virulence in *Ustilago maydis* [32–35] and *Mycosphaerella graminicola* [36].

The present study was initiated following an *in silico* analysis of the *N*- and *O*-glycosylation pathway components of the tomato pathogen *F. oxysporum* f.sp. *lycopersici*. As a result, a family of seven members of genes presumably encoding for *N*-acetyl glucosamine transferases (Gnts) was identified. Targeted gene disruption generated a double knock out mutant lacking a Golgi-localized Gnt, that displayed altered physico-chemical cell wall properties indicating severe structural changes, and a significant decrease in virulence on tomato plants. These conclusions were corroborated by functional complementation of the deletion mutant. This work opens the question to advance in the characterization of the *N*- and *O*-glycosylation as key enzymes decorating the outer cell surface of fungal plant pathogens. 

## Materials and Methods

### Fungal isolates, culture conditions and treatments

*F. oxysporum* f.sp. lycopersici wild type strain 4287 (race 2) was obtained from J. Tello, Universidad de Almería, Spain, and stored at -80°C with glycerol as a microconidial suspension. The pathotype of the isolates was periodically confirmed by plant infection assays. For extraction of DNA and microconidia production, cultures were grown in potato dextrose broth (PDB) (Difco) at 28°C as described previously [37]. For inhibition assays in axenic cultures freshly obtained microconidia from the wild type, the Δ*gnt2* mutant resistant to hygromycin (Hyg^R^) [38] and the cΔ*gnt2* complemented strain resistant to phleomycin (Phl^R^) [39] were transferred on 1.5% (w/v) agar plates of synthetic medium (SM) [37] containing 1% (w/v) glucose. For phenotypic analysis of colony growth, water droplets containing 5 x10^3^, 5 x10^2^ or 50 freshly obtained microconidia were spotted onto SM plates containing the indicated compounds. Plates were incubated at 28 °C for 3 days, or at 35°C for 6 days for heat stress assessment, before being photographed. For determination of sensitivity to cell wall-degrading enzymes, germlings were incubated with protoplasting enzyme (Glucanex G100, Denmark) at 30 °C, and protoplast release over time was determined microscopically as described previously [40]. To test sensitivity to Brefeldin A (BFA, Sigma) treatment, germlings grown for 12 h in PDB were washed with sterile water and incubated for 5 min in the presence of BFA (dissolved in ethanol) at 100 g mL^-1^ final concentration. Cells were then subjected to fluorescence and light microscopy analyses. 

### Nucleic acid manipulations and cloning

Total RNA and genomic DNA (gDNA) were extracted from *F. oxysporum* mycelium according to previously reported protocols [41–43]. Southern analyses and probe labelling were carried out as described previously [37] using the non-isotopic digoxigenin labelling kit (Roche Applied Science). *F. oxysporum* cDNA from duplicated genes FOXG_12436/FOXG_14101 including the complete ORFs, was amplified from total RNA using primers gnt2-31N corresponding to the eight initial codons of the ORF plus the *Nsi* I restriction site and an additional cytosine, and gnt2-33X corresponding to the reverse complement of the last eight codons of the ORF plus the *Xma* I restriction site and an additional cytosine (Table 1), derived from *F. oxysporum* genome sequence database (http://www.broad.mit.edu/annotation/genome/fusarium_group). The resulting amplified band was cloned into pGEMT vector (Promega, Madison*-*WI) and then subcloned in frame into the appropriate sites of the *Aspergillus nidulans* vector p1902 (kindly provided by Dr. M.A. Peñalva, CIB-CSIC, Spain), resulting in a GFP::Gnt2 fusion protein under the transcriptional control of the *gpdA* promoter and terminator. Sequencing of both DNA strands of the obtained clones was performed at the Servicio de Secuenciación Automática de DNA (Universidad de Córdoba, Spain) using the Dyedeoxy Terminator Cycle Sequencing Kit (Applied Biosystems, Foster City-CA) on an ABI Prism 377 Genetic Analyser apparatus (Applied Biosystems, Foster City-CA). DNA and protein sequence databases were searched using the BLAST algorithm [44] at the National Centre for Biotechnology Information (Bethesda, MD).

**Table 1:** Oligonucleotides used in this study.

**NaName**	**Sequence (5´→ 3´)**	**Position AT**	**Experimental use**
FOXG_12436F	GTTCGACATAAGGATAATACGGA	+350 (s)	RT-PCR
FOXG_12436R	TATTTCGGAGCCCAGATACTTG	+824 (as)	RT-PCR
chs V-3	ACAGCTCCAACGAACTCT	2910 (s)	Fungal quantification
chs V-26	GGAGGTACTTGGTCATGT	3402 (as)	Fungal quantification
tomQB-1	CCTCATCAACCAATCCTCCAA		Fungal quantification
tomQB-2	TCATTCACAACAACTCCAGGG		Fungal quantification
Actin-1	GAGGGACCGCTCTCGTCGT	898 (as)	RT-PCR
Actin-2	GGAGATCCAGACTGCCGCTCAG	674 (s)	RT-PCR
gnt2-sceIF	tagggataacagggtaatCCTCGTGAGTTTATCCAGCAG	-821 (s)	Delsgate disruption vector/ probe
gnt2-sceIR	attaccctgttatccctaCCCAGAAATCCAACAAGATAGG	+2030 (as)	Delsgate disruption vector
gnt2-attB1	ggggacaagtttgtacaaaaaagcaggctaaCAGGTACTCGCTATTGGTCAC	+166 (as)	Delsgate disruption vector
gnt2-attB2	ggggaccactttgtacaagaaagctgggtaGACTTCCAAATGAAACGCAAGG	+1032 (s)	Delsgate disruption vector
gnt2-3	AGTGAAGTTGTTGATTTTTGGTGG	+1461 (as)	Complementation
gnt2-7	GTGATCCTCTCGACGCAGAC	-1201 (s)	Complementation
gnt2-8	CTATTCAGCTACCTGCGCCAT	-232 (as)	Complementation/Probe
gnt2-18B	cgcggatccATGATAGGTGTCGCCCGATTA	+1 (s)	*gnt2* ORF amplification
gnt2-19S	acgcgtcgacCTAGTTCAGCTGCAGATTTCC	+1110 (as)	*gnt2* ORF amplification
gnt2-31N	catgcatATGATAGGTGTCGCCCGATTACTC	1 (s)	*gnt2* ORF amplification
gnt2-33X	ccccgggCTAGCTGCAGATTTCCTTGCGTTTCAT	+1110 (as)	*gnt2* ORF amplification
gpdA-15B	AATAGTGGTGAAATTGATCGTGT	gpdA Promoter	GFP fusion
tripter-8B	TCGACCATCCGGTGCTCTG	gpdA Terminator	GFP fusion
chi3-5	TCTTGTCTCTTTTTCTTGTTCC		qRTPCR plant-defence
chi3-6	GCAGTATCATCACCAGCAGT		qRTPCR plant-defence
chi9-5	GCCTTCTTGTCACGATGTCA		qRTPCR plant-defence
chi9-2	CTCCAAGAATTCCGCAATACC		qRTPCR plant-defence
gluB-7	ATTCTGTTTATGCTGCGATGG		qRTPCR plant-defence
gluB-8	CTTTCTCGGACTACCTTCTTT		qRTPCR plant-defence
pr1-7	GCATCCCGAGCACAAAACTA		qRTPCR plant-defence
pr1-8	TGGTAGCGTAGTTATAGTCTG		qRTPCR plant-defence
efα1-1	TACTGGTGGTTTTGAAGCTGG		qRTPCR plant-defence
efα1-2	AACTTCCTTCACGATTTCATCA		qRTPCR plant-defence

Italics and lower case indicate restriction sites and nucleotide sequences added for cloning purposes. Positions are referred to the start codon, (+) downstream or (-) upstream of ATG. Orientation is indicated, (s) sense, (as) antisense.

### Quantification of *F. oxysporum* biomass during plant infection

Plant roots were maintained immersed in microconidial suspensions of the different strains (5 x 10^6^ microconidia mL^-1^), for five days. To avoid amplification of gDNA from external fungal mycelium that had not penetrated the roots, only the stems were collected for DNA analysis 5 plants were used per treatment. Real-time PCR assays for the quantification of fungal gDNA from infected stems were performed using primer pair chs V-3 and chs V-26 (Table 1). Reaction mixtures contained 7.5 μL of FastStart Essential DNA Green Master (Roche Diagnostics), 300 nM of each primer and 60 ng of total DNA extracted from stems in a final volume of 15 L. Three simultaneous replicated amplifications were carried out for each DNA sample, using 15-μL aliquots from a 50-μL mixture. Amplification reactions were performed in 96-well microtitre plates (Bio-Rad). PCRs were performed in an iCycler apparatus (Bio-Rad) using the following cycling protocol: an initial step of denaturation (5 min, 94 °C) followed by 40 cycles of 30 s at 94 °C, 30 s at 62 °C, 45 s at 72 °C and 20 s at 80 °C for measurement of the fluorescence emission. After this, a melting curve programme was run for which measurements were made at 0.5 °C temperature increases every 5 s within a range of 55-95 °C. The DNA concentration of each sample was extrapolated from standard curves, which were developed by plotting the logarithm of known concentrations (10-fold dilution series from 100 ng to 1 ng/15 μL reaction) of *F. oxysporum* gDNA against the Ct values. In order to normalize the amplification conditions of the serially diluted DNA samples, 100 ng of DNA from non-inoculated plants were added to each sample in the dilution series. Additionally, tomato gDNA concentration was extrapolated from standard curves developed by plotting the logarithm of known concentrations (10-fold dilution series from 200 ng to 1 ng/15 μL reaction) of plant gDNA against the Ct values, using a primer pair corresponding to *Solanum lycopersicum tomQB* gene (β, 1-3 glucanase) (Table 1). Graphs represent the amount of fungal gDNA relative to 100 ng tomato gDNA. The experiment was repeated three times using independent infected tissues. Data were analysed with the software SPSS 15.0 for Windows® (LEAD Technologies, Inc.). ANOVA was performed and the Duncan *post hoc* test was executed to assess the differences among treatments within each day at *P* ≤ 0.05. 

### Quantitative RT-PCR of defence-related genes

Real-time RT-PCRs were performed in an iCycler apparatus (Bio-Rad) using 7.5 μL FastStart Essential DNA Green Master (Roche Diagnostics), 6.9 μL of cDNA template and 300 nM of each gene-specific primer (Table 1) in a final reaction volume of 15 μL. All primer pairs amplified products of 200-250 bp. The following PCR program was used for all reactions: an initial step of denaturation (5 min, 94 °C), followed by 40 cycles of 30 s at 94 °C, 30 s at 60 °C, 30 s at 72 °C and 20 s at 80 °C for measurement of the fluorescence emission. A melting curve program was run for which measurements were made at 0.5 °C temperature increments every 5 s within a range of 55-95 °C. Each sample reaction was performed in duplicate for each gene assay. Relative levels of the RT-PCR products were determined using the DDCt method [45]. Ct values were normalized to the Ct value of the elongation factor (*EF*α*1*) housekeeping gene. Normalized transcript levels of each gene in infected samples were compared with levels in non-inoculated samples. The experiments were repeated three times with independent infected tissues. Data were analysed with the software SPSS 15.0 for Windows® (LEAD Technologies, Inc.). ANOVA was performed to assess differences among treatments for each gene at *P* ≤ 0.05.

### Targeted gene replacement and complementation

Simultaneous targeted replacement of the duplicated *FOXG_12436/FOXG_14101* alleles (*gnt2*) was performed using the DelsGate technique [46]. The 5´and 3’ *gnt2* genomic flanking sequences, were obtained by PCR amplification of wild type gDNA, and the resulting 1025 bp and 1020 bp fragments, 5´and 3´ respectively, were cloned into pDONR vector containing the Hyg^R^ cassette (Figure S1A). For each transformation, the *Sce* I lineal DNA deletion construct (6000 bp), was introduced into protoplasts of wild type strain 4287 as reported previously [37]. Complementation of the Δ*gnt2* mutant was achieved by reintroducing the *gnt2* wild type allele obtained by DNA amplification using primer pair gnt2-7/gnt2-3 (Table 1), and co-transformation with the Phl^R^ cassette as selective marker. In all cases, Hyg^R^ or Phl^R^ resistant transformants were selected and the homologous recombination or complementation events were confirmed by Southern analysis of gDNA using the indicated probe (Figure S1B).

### Subcellular fractionation

Golgi-enriched fractions were isolated from fungal mycelia grown for 14h on PDB by ultracentrifugation on discontinuous sucrose gradients as previously described [47] with some modifications. Cells were thoroughly washed with water, disrupted by freezing liquid nitrogen and resuspended in 1 mL ice-cold HM buffer containing 10 mM HEPES, 1 mM MgCl_2_ pH 7.5, 1 mM PMSF and 1% protease inhibitor cocktail (Sigma). The cell homogenate was centrifuged at 4 °C during 5 min at 1,000 *g* to remove un-lysed cells. The supernatant was recovered and centrifuged at 4 °C during 10 min at 10,000 *g* to generate a pellet, corresponding to endoplasmic reticulum enriched fraction (P10) that was resuspended in 350 μL ice-cold HM and stored at -20 °C for further analyses, and a supernatant (S10) that was loaded on a 4 mL sucrose step gradient (26 to 54%). Sucrose solutions were prepared in HM buffer and the gradient column was incubated over night at 4 °C before use. Gradient was subjected to centrifugation at 4 °C for 90 min at 160,000 *g* in a SW50.1 rotor (Beckmann Coulter, Fullerton, CA) and 14 fractions of approx. 350 μL each were collected from the top of the gradient. The remaining pellet (P160) was resuspended in 350 μL ice-cold HM and stored at -20 °C for further analyses. Sucrose concentration of each fraction was measured using a refractometer (Atago Co., LTD). Aliquots of each fraction were mixed with SDS sample buffer and proteins were resolved by SDS-PAGE and detected by immunoblotting using the anti-GFP anti-body (Roche) or anti-Vps10p (Life Technologies). Guanidine diphosphate (GDP) hydrolysis and NADPH cytochrome c reductase assays were used to detect Golgi and endoplasmic reticulum enrichment, respectively, in the different fractions. Hydrolysis of GDP (GDPase) was measured as previously described [48] with some modifications. Five-μL aliquots of each fraction were mixed with 20 μL of reaction buffer containing 0.2 M imidazole pH 7.5, 10 mM CaCl_2_, 0.1% Triton X-100 and 2 mM GDP. The reactions were incubated in 96-well plates at 30 °C for 30 min and stopped by transferring them to ice and adding 2 μL of 10% SDS. Finally, 40 μL of water and 140 µL of AMES reagent (1:6 mixture of 10% ascorbic acid and 0.42% ammonium molybdate in 1 N sulfuric acid) were added to each well and the reactions were incubated at 42 °C for 20 min. GDPase activity of each fraction was determined as the absorbance at 660 nm.

To determine NADPH cytochrome c reductase activity, 10 µL aliquots of each fraction were assayed using the cytochrome c reductase (NADPH) Assay Kit (Sigma) following the manufacturer’s instructions, except that reactions were scaled to a final volume of 224 µL.

### Staining of Golgi complexes

For visualization of Golgi compartments, germlings from *Fusarium* were stained using the selective fluorescent sphingolipid BODIPY-TR (Molecular Probes) [49]. Aliquots containing 10^5^ microconidia from the different strains were inoculated on 1 % agarose plates containing 0.5 % casamino acids, and incubated at 28 °C for 14 h before treatment at RT with 2.5 % glucanex during 10 min. After washing three times with Hanks´ Balance Salt Solution (sodium chloride 8 g L^-1^; potassium chloride 0.4 g L^-1^; potassium phosphate monobasic 0.06 g L^-1^; glucose 1 g L^-1^; sodium phosphate dibasic 0.048 g L^-1^; magnesium sulphate 0.098 g L^-1^; calcium chloride 0.14 g L^-1^; sodium bicarbonate 0.35 g L^-1^) containing 10mM HEPES pH 7.4 (HBSS/HEPES), samples were stained during 30 min at 4 °C with 5 μM BODIPY-TR complexed with defatted BSA. Following three washes with HBSS/HEPES samples were flooded with liquid SM and further incubated at 28 °C during 60 min. Fixation of samples was achieved by treatment during 40 min at RT with fixation solution (50 mM phosphate buffer pH 7.0; 3.7 % formaldehyde; 0.2 % Triton X-100), followed by two washes with PBS buffer containing 0.0125 mM manganesium chloride and 0.0125 mM calcium chloride. Finally, samples were observed under optical and fluorescence microscopy.

### Phylogenetic analysis

Amino acid sequences were aligned using the CLUSTALW algorithm [50] with the Bioedit 7.0.0 program [51] and cleaned by GBlocks v0.91b [52]. The PHYML 3.0 program [53] was used to perform a 1,000 nonparametric bootstrap phylogenetic analysis of the resulting alignment with the maximum likelihood method after optimization of the settings by the MODELGENERATOR program, version 0.85 [54]. Phylogenetic relationships among sequences were depicted in a phylogenetic tree constructed using *MEGA* version 4 [55].

### Alcian Blue staining

Alcian Blue binding assay was carried out using the method of Herrero et al. [48]. A series of solutions containing different amounts of Alcian Blue (Sigma) was prepared in HCl 0.002 N, and the optical density at 620 nm (OD_620_) of each solution was determined. A standard curve was plotted of the OD values versus amounts of Alcian Blue. To quantify Alcian Blue bound to the cell surface, fresh aliquots containing 5 x 10^8^ microconidia were centrifuged, and the cells were washed with 0.002 N HCl, resuspended in 0.025 % Alcian Blue (w/v), incubated 20-30 min at RT, and then centrifuged for 2 min to pellet the cells. The OD_620_ of the supernatant was measured and the amount of Alcian Blue was determined by using the standard curve. The percentages of dye bound to the cells were calculated from the total values. 

### Cell surface labelling with GS II-FITC lectin and flow cytometry analysis

The lectin GSII from *Grifonia simplifolia* labelled with Fluorescein isothiocyanate (FITC) was used in this study due to its specific binding capacity to terminal *N-*acetylglucosamine residues [56–58]. Lectin GSII-FITC stock was prepared at 1 mg mL^-1^ in 10 mM phosphate buffer, containing 15 mM NaCl and 50 mM CaCl_2_. Aliquots containing 10^7^ fresh microconidia or germlings grown for 3 h on PDB were collected, centrifuged at 5,000 g for 5 min, washed twice with water and twice with labelling buffer (50 mM phosphate buffer pH7.5, containing 150 mM NaCl, 1 mM CaCl_2_, 0.5 mM MgCl_2_ and 0.1 mM MnCl_2_) and then resuspended in 180 μL of the same buffer. Samples were stained by adding 20 μL GS II-FITC (F-2402-2, EY laboratories, USA) at final concentration 0.1 mg mL^-1^ and incubated for 30 min at 28 °C, 170 rpm, in the dark. After washing twice with labelling buffer, samples were resuspended at 5 x 10^6^ cells mL^-1^. The fluorescent emission of cell surface labelling was determined by flow cytometry analysis. Fluorescence intensity measurements were performed by flowing labelled cells at a rate of 200-300 sec^-1^, excited at 488 nm, through a FACScan (BD Biosciences, San Jose, CA) equipped with an Innova 90 argon laser at the Department of Cell Biology, Immunology and Physiology (University of Cordoba, Sian). The green emission (550 nm) was collected by a FL1 detector. For each strain, a total number of 20,000 FITC event data files were collected and analysed with Lysis II on a Hewlett-Packard 340 computer. The data of independent fluorescence emission were processed as frequency distribution histograms and the flow cytometer light scattering measurement, corresponding to the size and shape of cell population, were represented by a two-parameter, forward and side light scatter histogram, FSC and SSC, respectively. 

Single-cell and aggregated conidia in the whole population were identified by plotting all FITC events and representing their relative fluorescence intensity (FL1 channel) vs. their lineal fluorescence intensity (auxiliary channel) values. Single cell populations were classified as those positive events (H3) that fell below the relative fluorescence value of 10^1^ considered as a single cell maximal emission, as deduced from the flat slope observed in the strain graphs. Thus, aggregated cell population (H4) was identified as scattered values showing higher lineal fluorescence intensities. This approach was further supported by morphological analyses using light scattering assessments. All experiments were performed at least three times.

### Cell wall material preparation and fractionation

Glycans linked to cell wall glycoproteins were extracted from fungal mycelium, grown for 3 days in minimal medium containing 1 % sucrose as the carbon source, following the previously described protocol [59] with minor modifications. Freeze-dried mycelium (3 g) was ground using an IKA 10A grinder and *O*-linked glycans were released after four consecutive extractions at 20 °C with 40 mL 0.1 M NaOH containing 0.3 M NaBH_4_, with shaking during 8 h. After centrifugation at 10.000 *g* for 20 min at 10 °C, the supernatants were combined and two volumes of absolute ethanol were added. The samples were incubated at 4 °C over night and the precipitate was collected by centrifugation, resuspended in 30 mL of water and dialyzed (3.5 kDa cut-off) against distilled water for 4 days. The resulting insoluble (F1) and water-soluble fractions were freeze-dried and the latter was again fractionated by ethanol-water 1:1 (v/v) solubility followed by centrifugation. The resulting insoluble fraction (F2) was freeze-dried, and the soluble fraction, containing *O*-linked glycans, was dialyzed against distilled water for 8 h and freeze-dried (F3). *N*-linked glycans were released from the solid residue obtained after the fourth treatment with 0.1 M NaOH/0.3 M NaBH_4_, by four consecutive extractions with 40 mL 1 M NaOH at 20 °C with shaking during 8 h, followed by centrifugation at 15,300 *g* for 20 min at 10 °C. The resulting solid residue, containing the β-glucan-chitin complex (F4), was dialyzed (12 kDa cutoff) and freeze-dried, and the supernatants were subjected to precipitation with absolute ethanol followed by consecutive solubilisation with water and ethanol: water 1:1 (v/v), as described above. The resulting water and ethanol: water insoluble fractions (F5 and F6, respectively), as well as the ethanol: water soluble fraction, containing *N*-linked glycans (F7), were dialyzed against distilled water for 8 h, and freeze-dried. Finally, the dry-weight of each fraction was quantified, and the amount of *O*- and *N*-linked glycans was determined relative to the cell wall total dry weight (fractions F1 to F7). Experiments were repeated three times. 

### Aggregation and cell-to-cell adhesion assays

Spore and hyphal aggregation ability was assessed at different pH values following a method previously described [60]. Briefly, 50 mL of liquid SM were adjusted to pH 2.0, 3.5 or 6.0, inoculated with 10^6^ microconidia mL^-1^, incubated at 28 °C and 80 rpm for 5 to 7 h. To quantify spore aggregation, aliquots of each culture were observed under a light microscope and 15-20 random pictures of every strain were taken, resulting in 300-500 cell counts. To avoid errors caused by non-spore particles, all images were controlled and false measurements erased. In parallel, GSII-FITC labelled cells were observed under the fluorescence and light microscope, and a number of random pictures were taken, resulting in 300-500 cell counts. 

### Optical, fluorescence and transmission electron microscopy

For optical and fluorescence microscopy analyses cell aliquots were embedded in 1% agarose blocks, and observed using the Nomarsky technique or the appropriate filter set, respectively, in a Zeiss Axio Imager M2 microscope (Carl Zeiss MicroImaging GmbH, Göttingen, Germany). Images were captured with an Evolve Photometrics digital camera using the Axiovision 4.8 software. Images were processed using Adobe Photoshop C5 (Adobe Systems, Mountain View, CA, USA).

For transmission electron microscopy (TEM), germlings grown on PDB for 14 h at 28 °C and 150 rpm were initially fixed overnight at 4 °C, in a mixture of 2.5 % glutaraldehyde and 2 % paraformaldehyde in 0.1 M sodium cacodylate buffer pH 7.0, then washed in buffer and post-fixed in 1 % osmium tetroxide at 4 °C. After dehydration in an ethanol series, the samples were treated with propylene oxide and embedded in EMBed 812. After curing, the blocks were sectioned with a thickness of about 80 nm in an ultramicrotome and mounted on Cu grids. The samples were stained in 2 % aqueous uranyl acetate for 2 min at 37 °C, and then transferred to Reynold’s lead citrate for 3 min at room temperature. Micrographs were obtained using a Philips CM 10 electron microscope.

### Plant and animal infection assays

Tomato root inoculation assays were performed as described [37], using 2-week-old tomato seedlings (cultivar Monika, seeds kindly provided by Syngenta, Spain) and *F. oxysporum* strains, by immersing the roots in a suspension of 5 × 10^6^ spores mL^-1^ for 30 min, planted in vermiculite and maintained in a growth chamber. Ten plants were used for each treatment. Severity of disease symptoms and plant survival was recorded daily for 30 days as previously described [61]. 

*Galleria mellonella* larvae in the final larval stage were obtained from the company Animal Center S.C.P. (Valencia, Spain), and inoculated as previously described [62]. Fifteen larvae per treatment between 0.2 to 0.3 g in weight were employed in all assays. A Burkard Auto Microapplicator (0.1-10 µL; Burkard Manufacturing Co. Limited, Hertfordshire, UK) with a 1 mL syringe was used to inject 8 µL of a microconidial suspension, containing 1.5 x 10^5^ spores resuspended in sterile phosphate-buffered saline (PBS), into the hemocoel of each larva through the last left proleg. The area was cleaned using an alcohol swab before injection. Larvae injected with 8 µL PBS served as controls. After injection, larvae were incubated in glass containers at 30 °C, and the number of dead larvae was scored daily. Larvae were considered dead when they displayed no movement in response to touch.

The Mantel-Cox method was used to assess statistical significance of differences in survival among groups. Data was plotted using Graph Pad Prism software version 4 for Windows. Differences showing a P value < 0.05 were considered significant. Experiments were repeated three times with similar results. Data presented are from one representative experiment.

## Results

### Identification and sequence analysis of glycosylation-decorating enzymes in the Fusarium oxysporum genome

Since glyco- structures are excellent targets for host recognition in animal and plant model systems, we conducted an *in silico* analysis of the *N*- and *O*-glycosylation pathway components of the tomato pathogen *F. oxysporum* f.sp. *lycopersici* strain 4287 genome sequence database. The results of the protein blast search revealed that the *F. oxysporum* genome contains homologous sequences to 49 *S. cerevisiae* genes involved in *O*- and *N*- protein glycosylation. Remarkably, we detected the existence of seven α-1-4,*N-*acetylglucosamine transferase paralogs and the apparent absence of 17 orthologs to yeast genes in this pathogenic fungus (Table S1). We were conservative in our selection, requiring *E* values ≤ than e^-9^ to consider candidate sequences as putative *F. oxysporum* protein homologs. 

The family of seven members encoding for N-acetyl glucosamine transferases (all named *gnt* in this work) was further studied: FOXG_12436 (*gnt2*) and FOXG_14101 (*gnt5*) are identical copies located within duplicated genomic regions on chromosomes 3 and 6, respectively (here after *gnt2*); FOXG_01495 (*gnt1*) on chromosome 5; FOXG_12874 (*gnt3*) and FOXG_12897 (*gnt4*) both on chromosome 9; FOXG_14149 (*gnt6*) and FOXG_16408 (*gnt7*) both on chromosome 14. The deduced amino acid sequences from FOXG_12436, FOXG_14101 and FOXG_14149 genes presented 31-33% identities to *S. cerevisiae* GNT1 [21], a fungal enzyme belonging to the glycosyl transferases family 8 that catalyses the addition of *N-*acetyl-D-glucosamine to mannose side chains by high mannose N-glycan synthesis [10].

Cloning and sequencing of the complete cDNAs from *gnt2, gnt4, gnt6* and *gnt7* allowed manual curing of the genomic sequences in the *F. oxysporum* genome database using BLAST and the non-redundant database of NCBI. *Gnt2* consists of an ORF of 987 bp organized in 3 exons interrupted by 2 introns: exon I starts 264 nucleotides upstream from the annotated start codon and the first intron of 81 bp is 376 bp downstream of this putative start codon. Exon II is interrupted by a previously not annotated 58 bp intron, causing a frame shift and giving rise to a new exon III which ends 5 nucleotides upstream from the previously annotated stop codon, resulting in a 328 amino acid protein with a predicted trans-membrane domain between amino acids 12 to 34. *Gnt4* contains an ORF of 1023 bp organized in 3 exons interrupted by 2 introns. Intron 2 is 55 bp shorter than in the annotated version, followed by a new exon 3 of 63 bp and a stop codon 158 bp upstream of the previous annotation, resulting in a new open reading frame that codes for a 341 amino acid protein with a predicted trans-membrane domain between amino acids 7 to 24. *Gnt6* contains an ORF of 990 bp organized in 3 exons interrupted by 2 introns. Exon I starts 267 nucleotides upstream from the annotated start codon and the first intron of 81 bp is 379 bp downstream of this putative start codon, Exon II is interrupted by a new 58 bp-long intron, causing a frame shift and giving rise to a new exon III which ends 26 nucleotides upstream from the previous stop codon, resulting in a 329 amino acid protein with a predicted trans-membrane domain between amino acids 12 to 34. *Gnt7* contains an ORF of 1077 bp organized in 3 exons interrupted by 2 introns. The first intron is 126 bp shorter than the annotated one, giving rise to a 126 bp longer exon II and resulting in a 358 amino acid deduced protein.

*Gnt1* has an ORF of 1065 bp encoding a 354 amino acid polypeptide organized in 3 exons interrupted by 2 introns. The first intron of 75 bp is 415 bp downstream of the putative start codon, and the second of 53 bp is 54 bp upstream of the stop codon. The putative trans-membrane domain comprises amino acids 22 to 44. *Gnt3* has an ORF of 1269 bp encoding a 422 amino acid polypeptide organized in 3 exons interrupted by 2 introns. The first intron of 49 bp is 607 bp downstream of the putative start codon, and the second of 53 bp is 69 bp upstream of the stop codon.

**Figure 1:**
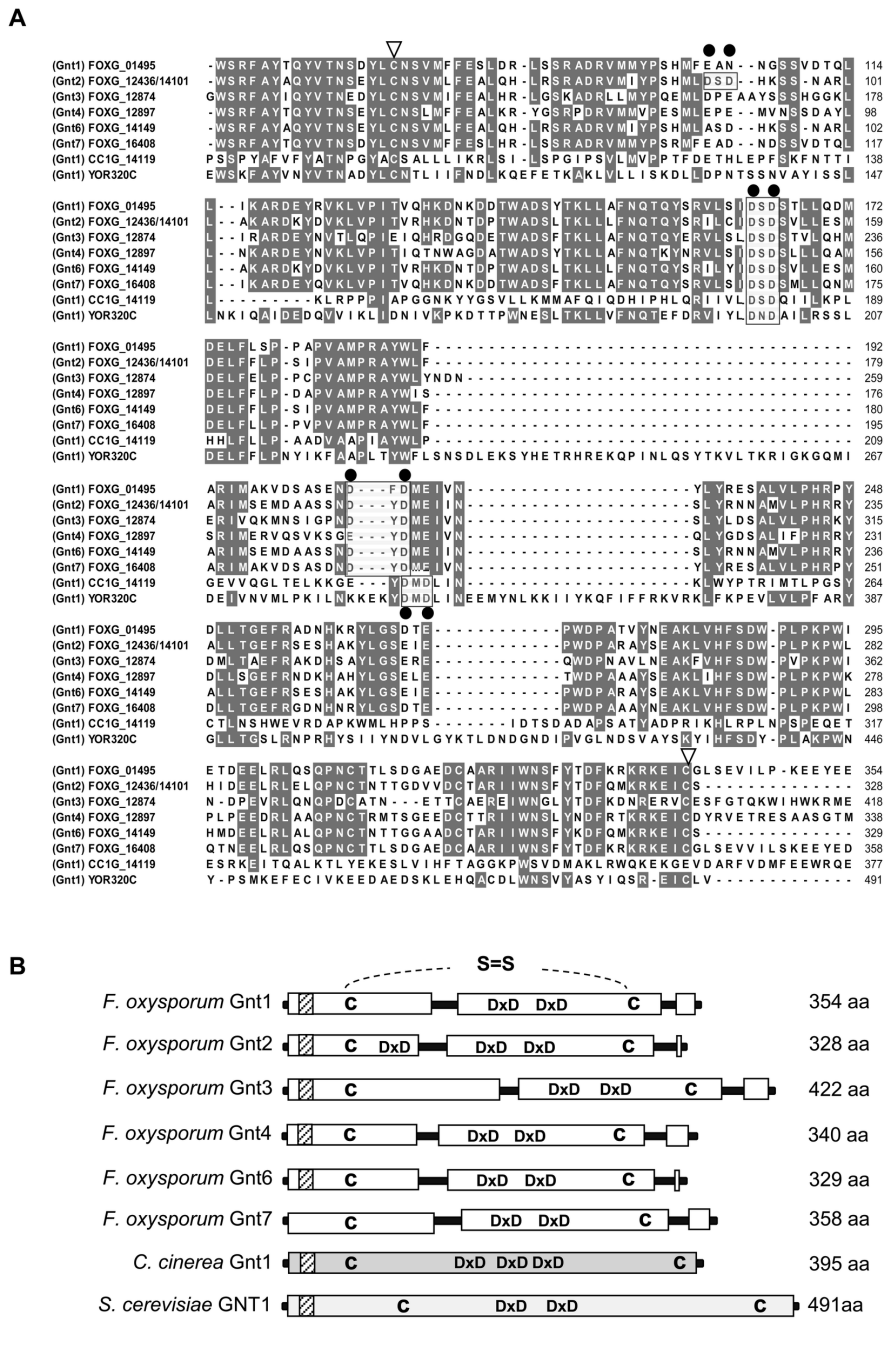
*Fusarium oxysporum* contains six putative *N-*acetyl glucosaminyltransferase genes. (**A**) Alignment of the conserved domains of six predicted Gnts encoded by *F. oxysporum* f.sp *lycopersici* genes FOXG_*01495* (Gnt1), FOXG_*12436/FOXG*_*14101* (Gnt2), FOXG_*12874* (Gnt3), FOXG_*12897* (Gnt4), FOXG_*14149* (Gnt6), and *FOXG-16408* (Gnt7), with the corresponding orthologs from *Saccharomyces cerevisiae* Gnt1 (YOR320c) and *Coprinopsis cinerea* Gnt1 (CC1G 14119). Identical amino acids are highlighted on a grey background. Conserved motifs: DXD triads (black rectangle), Asp residues involved in ion binding (filled black circle), and conserved Cys residues (open triangles) are indicated. Amino acid sequences were aligned using Clustal W. (**B**) Scaled diagrams with conserved features of *F. oxysporum* f.sp. *lycopersici* Gnt proteins. Positions of the conserved DXD domains, Cys residues (C), and transmembrane domains (dashed boxes) are indicated.

The deduced six proteins (Gnt1, Gnt2, Gnt3, Gnt4, Gnt6 and Gnt7) show an overall identity among each other ranging from 53 to 97 %. Alignment of the *F. oxysporum* f.sp *lycopersici* putative Gnts with other orthologs revealed identities of 31-33 % to S. cerevisiae GNT1 [21] and of 16-19 % to *Coprinopsis cinerea Ccgnt1* [16] (Figure 1A). All seven deduced proteins present the main features described for *N-*glycosyltransferases, located at highly conserved positions: a single pass trans-membrane domain (type II) near the N-terminus characteristic of a Golgi localized enzyme [63], and two/three DXD motifs essential for the coordination of the catalytic cations, most commonly Mn^2+^, and cysteine residues for establishment of disulfide bridges (Figure 1B).

### Gnt representation in multiple family members is unique for F. oxysporum

**Figure 2:**
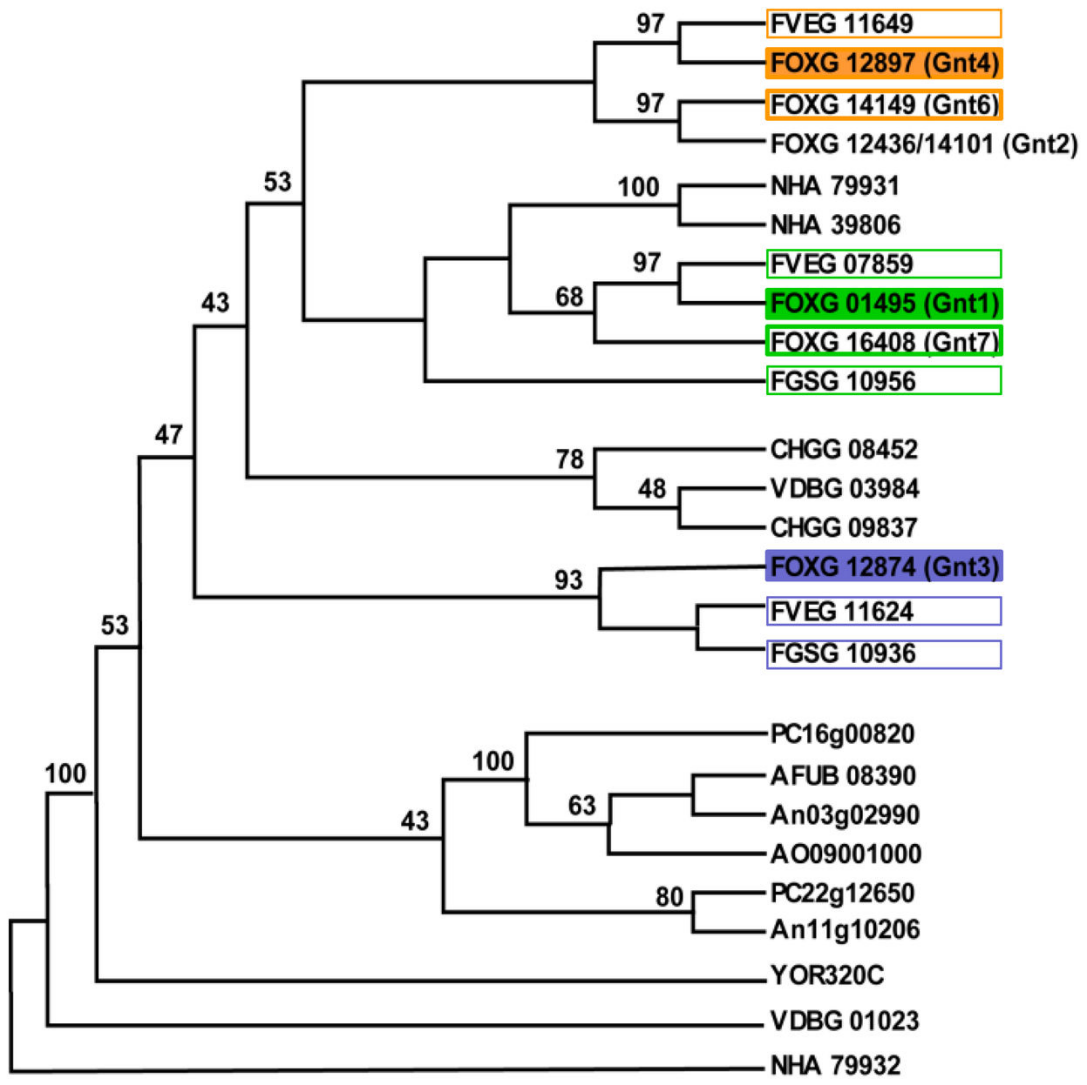
*gnt2* is part of a gene family expanded in the pathogenic fungus *F. oxysporum*. Phylogenetic tree constructed using the neighbour-joining method from Clustal W multiple-sequence alignment of 146 aa of GT8 proteins. Numbers at the branch points represent percentage bootstrap values based on 1,000 replicates. *F. oxysporum* (FOXG_01495; FOXG_12436/FOXG_14101; FOXG_12874; FOXG_12897; FOXG_14149; FOXG_16408)*, F. graminearum* (FGSG_10936; FGSG_10956*)*, F. verticillioides* (FVEG_07859; FVEG_11624; FVEG_11649*), *Aspergillus fumigatus* (AFUB_08390), *A. niger* (An11g10206; An03g02990), *A. oryzae* (AO09001000), *Chaetomium globosum* (CHGG_09837; CHGG_08452), *Saccharomyces cerevisiae* (GNT1, YOR320C), *Nectria haematococca* (NHA_39806; NHA_79931; NHA_79932), *Verticillium dahliae* (VDBG_01023; VDBG_03984), *Penicillium chrysogenum* (PC_16g00820; PC_22g12650). *These genes were manually cured as follows: FGSG_10956, 120 amino acids were added upstream of the annotated start codon; FVEG_11649, 78 amino acids were added upstream of the annotated start codon.

To investigate the representation of *gnt* genes among *Fusaria* and other fungal species, we performed an *in silico* search of the *Fusarium* comparative database (http://www.broadinstitute.org/annotation/genome/fusarium_group/) using the six deduced Gnt amino acid sequences identified in *F. oxysporum*. This search detected two or three orthologs in the majority of the species analysed, whereas *S. cerevisiae* contained only one (GNT1), indicating low genomic redundancy in all species, in contrast to the six genes located in seven loci at five different chromosomes in *F. oxysporum*. *F. oxysporum* Gnts were aligned together with other Gnts available at the National Centre for Biotechnology Information (http://blast.ncbi.nlm.nih.gov/Blast.cgi?PROGRAM=blastp) (Figure S2). The phylogram revealed that *F. oxysporum* Gnts are grouped in two clades, one containing Gnt1, Gnt2, Gnt4, Gnt6 and Gnt7 and the other containing Gnt3 (Figure 2). It is remarkable that four of the loci are located on three dispensable *F. oxysporum* f.sp. *lycopersici* chromosomes [64]. And thus, it could be assumed that genes *gnt1* (on chromosome 5), *gnt3* and *gnt4* (both on chromosome 9) were the ancestors of the other four genes which might be originated by genomic-duplication. This hypothesis is also supported by the number of orthologues found in the close related species *F. verticillioides* and *F. graminearum* (three and two genes, respectively).

### Targeted disruption of F. oxysporum gnt2 genes

To determine whether Gnts are necessary for pathogenicity of *F. oxysporum* f.sp. *lycopersici*, we performed double targeted replacement of the *gnt2* genes *FOXG_12436* and *FOXG_14101*. The disruption vector was introduced into wild-type protoplasts (Figure S1A), and homologous recombination events were confirmed by Southern analyses (Figure S1B). The wild type strain showed a 2 kb *Eco*R I/*Xho* I hybridizing band corresponding to *gnt6* plus a 3.5 kb double hybridizing band corresponding to the duplicated *gnt2* allele. The 3.5 kb band was replaced by a 7 kb fragment in the homologous integrative transformant #55, indicating that it has undergone simultaneous disruption of both *gnt2* alleles (*gnt2*) (Figure S1B). Transformant #46 displayed the original 3.5 and 2 kb *Eco*R I-*Xho* I hybridizing bands plus an additional band, indicating ectopic insertion of the disruption construct (Figure S1B).

Complementation of mutant Δ*gnt2* was done by cotransformation with the *gnt2* wild type allele and the Phl^R^ cassette as the selective marker. Cotransformants were identified by Southern analysis of gDNA digested with *Eco*R I and *Xho* I (Figure S1B). In the case of complemented strain 6 (cΔ*gnt2*), the hybridizing patterns allowed the verification of the integration events.

### Gnt2 is essential for virulence of Fusarium oxysporum

**Figure 3:**
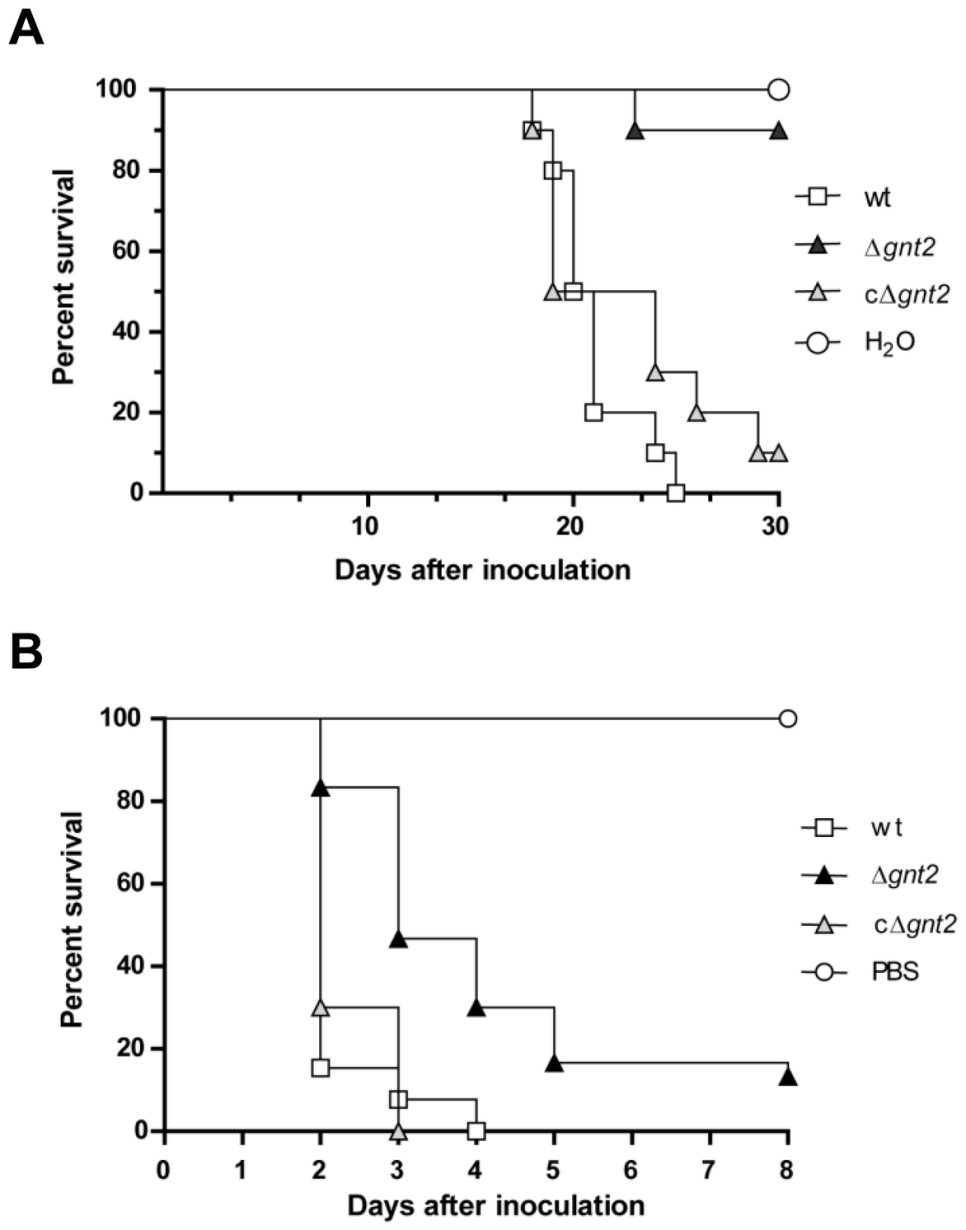
Gnt2 contributes to virulence of *F. oxysporum* on plant and animal systems. (**A**) Groups of ten plants (cultivar Monika) were inoculated by immersing the roots into a suspension of 5 x 10^6^ freshly obtained microconidia mL^-1^ of the wild type (wt), the Δ*gnt2* mutant and cΔ*gnt2* complemented strains, and planted in minipots. Percentage survival was recorded after different time points. All experiments were performed at least three times with similar results. The data shown are from one representative experiment. (**B**) Mantel-Cox plots of *Galleria mellonella* larvae survival after injection of 1.6 x 10^5^ microconidia of the indicated strains into the hemocoel and incubation at 30 °C. Data shown are from one representative experiment. Experiments were performed three times with similar results.

The role of Gnt2 in *F. oxysporum* f.sp. *lycopersici* virulence was determined by plant infection assays, performed by immersing the roots of 2-week-old tomato plants in microconidia suspensions of the fungal strains. Severity of wilt symptoms in plants inoculated with the wild type strain, the ectopic transformants (not shown) and the complemented cΔ*gnt2* strain increased steadily throughout the experiment, showing characteristic wilt symptoms 10 days after inoculation with most plants dead 8 days later. By contrast, plants inoculated with the Δ*gnt2* mutant showed a significant delay in symptom development with most plants alive (*P* < 0.0001) and healthy 25 days after inoculation (Figure 3A). Virulence experiments were performed three times with similar results. 

In order to examine the role of Gnt2 in *F. oxysporum* f.sp. *lycopersici* virulence on animals, we performed infection experiments using the greater wax moth *Galleria mellonella* as host, which has been recently described as an useful non-vertebrate infection model for studying virulence mechanisms of *F. oxysporum* on animal hosts [62]. Injection of microconidia of the wild type strain into the hemocoel of *G. mellonella* resulted in rapid killing of the larvae (Figure 3B). By contrast, the *gnt2* mutant showed a moderate but significant (*P* < 0.0001) reduction in killing. The complemented strain cΔ*gnt2* did not show significant differences in killing efficiency compared to the wild type strain.

**Figure 4:**
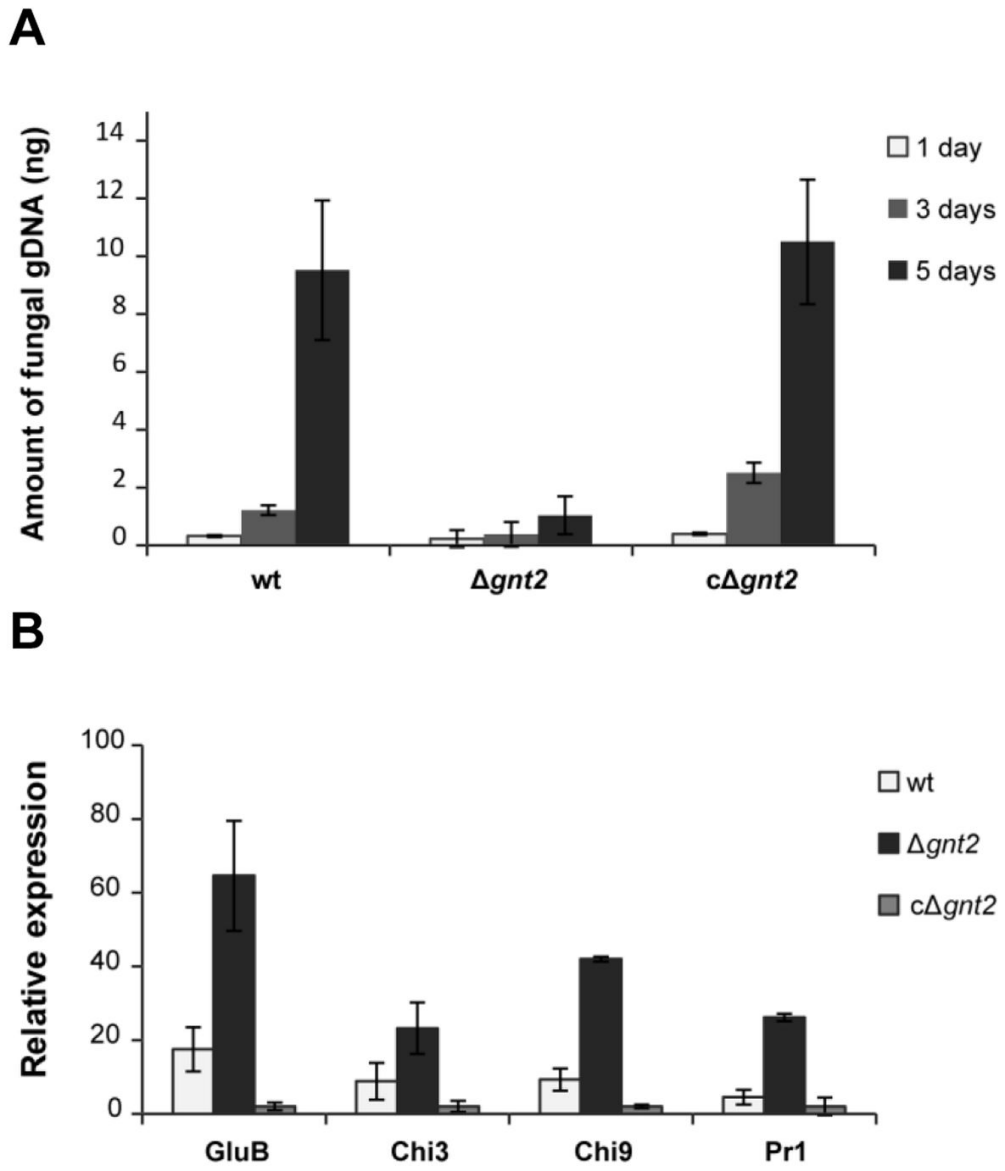
Δ*gnt2* mutants have reduced colonization ability and induce higher defence response of tomato plants. (**A**) Comparative analysis of fungal biomass using quantitative real-time polymerase chain reaction during disease progression caused by the wild type (wt), the Δ*gnt2* mutant and the complemented cΔ*gnt2* strains. Data represent nanograms of fungal DNA amplified from 100 ng of DNA extracted from infected stems. Each column represents the mean from three independent inoculation experiments with three replicates each. Standard error bars are indicated. (**B**) Expression of defence-response genes in tomato plants three days after inoculation with the indicated strains. Transcript abundance was determined by quantitative reverse transcriptase-polymerase chain reaction. Expression levels in each sample were normalized to the expression of the tomato *efα* gene and were calculated relative to the uninfected control plants by the DDCt method. Error bars indicate the standard error calculated from three independent inoculation experiments with three replicates each.

To determine the ability of the Δ*gnt2* mutant to colonize tomato plants, we performed quantification of specific fungal DNA within stems using real-time PCR. The amount of fungal biomass present in tomato stems increased during the course of infection in wild type-inoculated plants (up to 5 % of total DNA from infected tissues five days post-inoculation) (Figure 4A). A significant decrease in the amount of fungal DNA was observed in plants inoculated with the Δ*gnt2* mutant (up to 1 % of total DNA from infected tissues five days post-inoculation). These data suggest that Δ*gnt2* mutant colonizes the stem tissues with strong reduced efficiency in comparison with wild type strain. No significant differences were observed in complemented strain cΔ*gnt2* compared to wild type. No DNA amplification was observed in the un-inoculated controls (Figure 4A).

The plant defence reaction in response to infection with the wild type and non-virulent Δ*gnt2* strains was analysed by the quantification of transcript levels of defence-related genes encoding basic glucanase (*gluB*) [65], acidic chitinase 3 (*chi3*), basic chitinase 9 (*chi9*) [66] and pathogenesis-related protein 1 (*pr-1*) [65] in tomato plant roots at three days post-inoculation, using quantitative real-time RT-PCR (Figure 4B). The expression level of each defence-related gene was compared among plants infected with the wild-type strain, non-virulent Δ*gnt2* mutant, or the complemented strain cΔ*gnt2* as well as non-inoculated control plants, and referred to the relative levels of the constitutive reference gene *efα1* encoding elongation factor alpha 1 [67]. The expression levels of the four genes detected in plants inoculated with the mutant were significantly higher than those observed in plants inoculated with either the wild type or the complemented strains (Figure 4A). 

### *Δgnt2* mutant cell wall has altered physico-chemical properties

**Figure 5:**
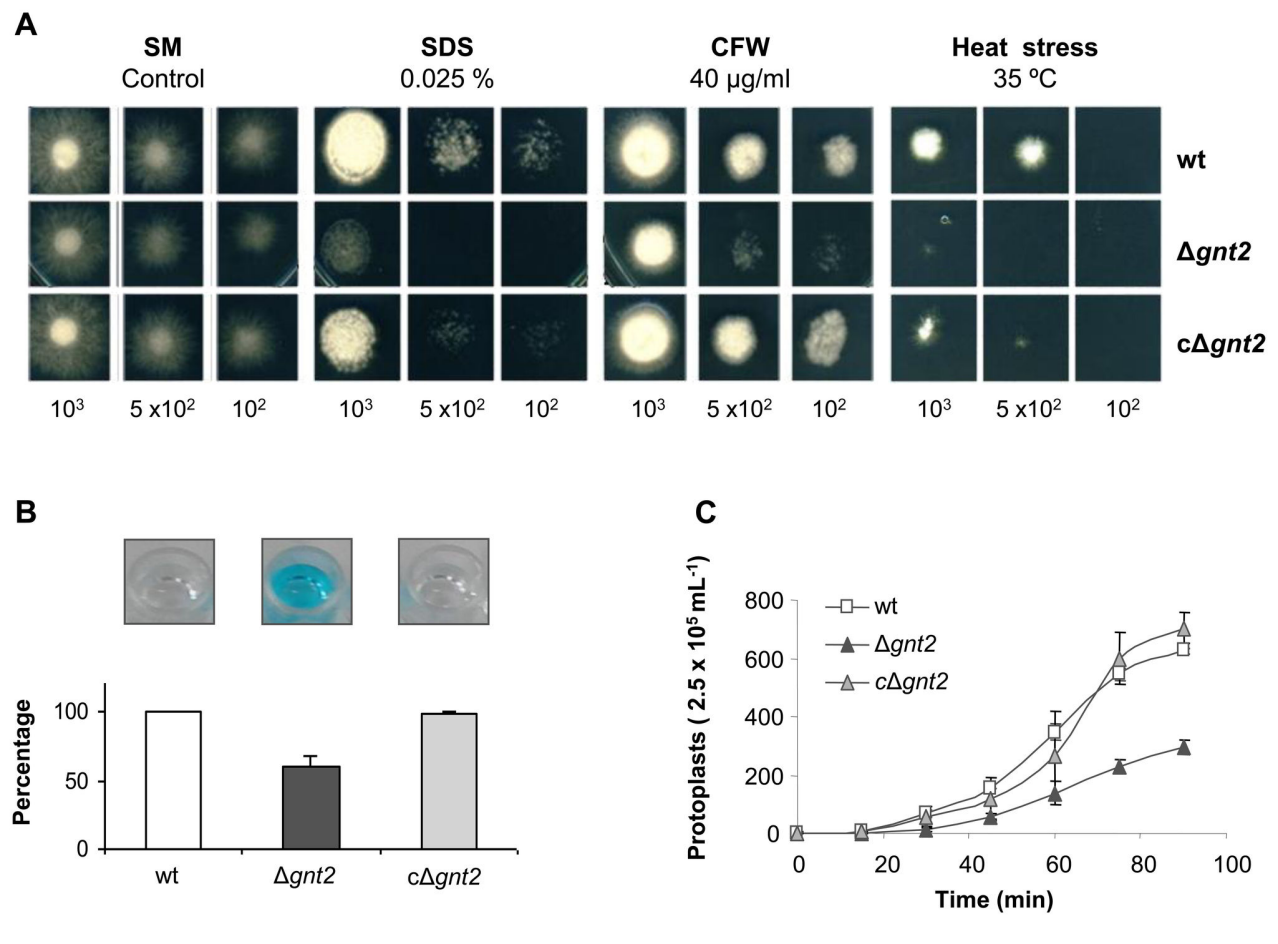
Gnt2 mutants show increased sensitivity to stress conditions. (**A**) Fungal colonies from the wild type (wt), the Δ*gnt2* mutant and the complemented cΔ*gnt2* strains grown for 3-4 days at 28 °C on SM plates containing Sodium Dodecyl Sulphate (SDS) or Calcofluor white (CFW), or on SM plates under heat stress conditions (120 h at 35 °C). The number of inoculated spores is indicated. (**B**) Alcian Blue binding affinity of *F. oxysporum* wild type (wt), Δ*gnt2* mutant and cΔ*gnt2* complemented strains. Microconidial suspensions (5 x 10^8^ mL^-1^) from the indicated strains were added to an Alcian Blue containing suspension (0.025% w/v), incubated 20 min at RT, and centrifuged. The ability to bind the dye was determined by measuring the absorbance at 620 nm of the remaining supernatant after centrifugation, and represented as % of Alcian Blue bound to the cells. Photographs above the diagram show the remaining colour in the supernatants of the different strains. (**C**) Sensitivity of *F. oxysporum* wild type (wt), Δ*gnt2* mutant and cΔ*gnt2* complemented strains to the treatment with 50 mg mL^-1^ Glucanex lytic enzyme. The graph shows the number of protoplasts released from each strain during incubation for the indicated time period in min. Bars indicate the standard error from three independent experiments.

The role of *F. oxysporum* Gnts on cell wall structure and integrity was investigated by determining the sensitivity to membrane or cell wall interfering agents and to heat stress conditions. As shown in Figure 5A, the Δ*gnt2* mutant exhibited higher sensitivity to Sodium Dodecyl Sulfate (SDS) and Calcofluor white (CFW), as well as to heat stress (35 °C), than the wild type strain. As expected, reintroduction of the wild type allele completely restored the resistance to the cell wall interfering agents tested, suggesting that these phenotypes may be a general consequence of compromised cell wall integrity.

The tetra-cationic compound Alcian Blue is able to detect negatively charged heteropolysaccharides, such as sulfated and carboxylated muco-polysaccharides and sialomucins (glycoproteins) in fungal cell surfaces. Reversible electrostatic bonds are formed between this cationic dye and the negative sites on the polysaccharides [68]. To further identify possible structural differences in the cell wall of the Δ*gnt2* mutant, microconidial suspensions were added to Alcian Blue solution and the absorbance of the remaining supernatant was measured at 620 nm. As shown in Figure 5B, the Δ*gnt2* mutant displayed a 33% decreased binding affinity to Alcian Blue compared to the wild type or the complemented strains. These phenotypes prompted us to further examine the cell wall properties of the Δ*gnt2* mutant. Germinated microconidia were exposed to a mixture of cell wall-degrading enzymes and the release of protoplasts was monitored over time. The extent of protoplast formation was followed by microscopic observation of the cell suspension at timed intervals and standardized to viable cells. As shown in Figure 5C, the Δ*gnt2* mutant showed dramatically decreased sensitivity to the activity of the protoplasting enzyme in comparison. 

### *gnt2* deficient mutant exhibits altered aggregation behaviour

**Figure 6:**
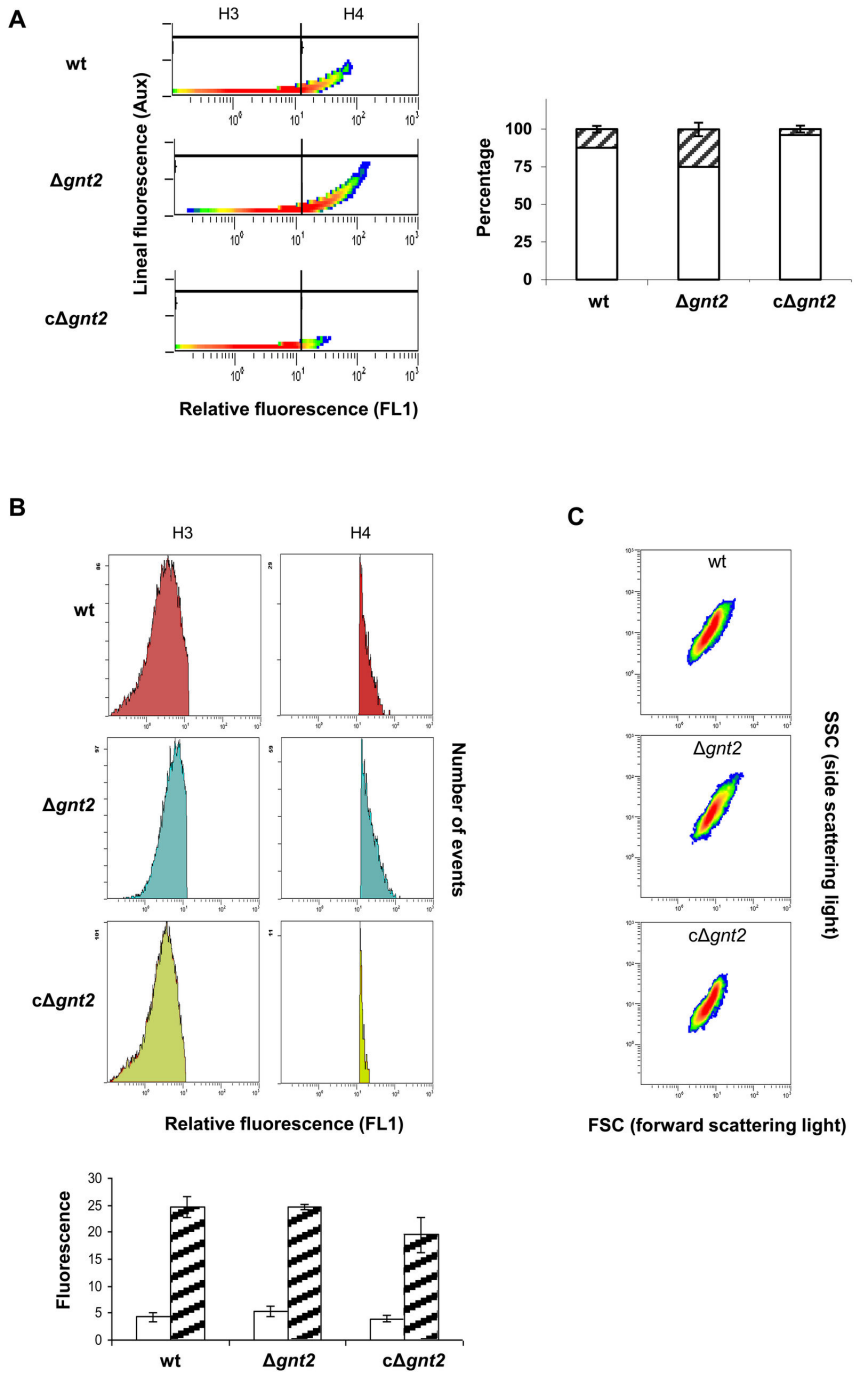
GS II-FITC lectin binding affinity of the indicated *F. oxysporum* strains. Intensity of fluorescence emitted by GS II-FITC bound to cell surface was measured for 20,000 events in microconidia from wild type (wt), Δ*gnt2* mutant and cΔ*gnt2* complemented strain, using flow cytometer separation and fluorescence detection. (**A**) Fluorescence analysis using the auxiliary channel adjusted to allow discrimination of single cell population (H3 in the histograms and white columns in the graph) from aggregated cells population (H4 and stripped columns). The percentages of each cell population are represented for the three strains. (**B**) Histograms showing relative fluorescence determined by FL1 channel for single cells (H3) or aggregated cells (H4) from the three strains. Columns in the graph represent the relative fluorescence medium values observed for single cells (white) and aggregated cells (stripped). The standard errors from three independent experiments are indicated. (**C**) Morphological analyses of cell populations from the different strains by cytometer light scattering detection. Forward and side scattered light shape (FSC and SSC, respectively) is represented.

**Figure 7:**
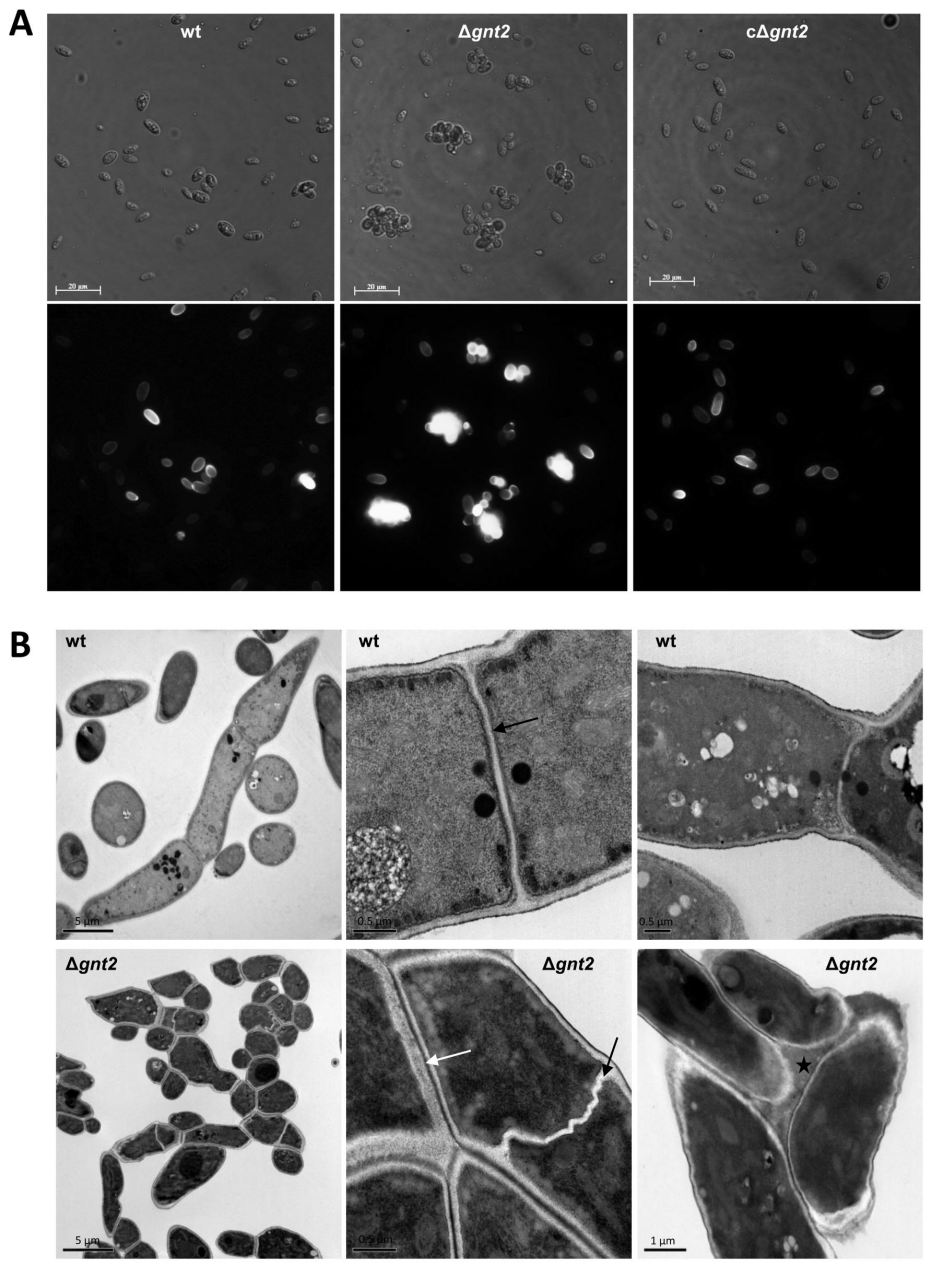
Deletion of *gnt2* gene results in increased cell aggregation and aberrant septum morphology. (**A**) Light (upper panels) and fluorescence (lower panels) micrographs of GS II-FITC labelled swollen spores (after 3 h incubation in PDB) from wild type (wt), Δ*gnt2* mutant and cΔ*gnt2* complemented strains. Scale bars, 10 µm. (**B**) Transmission electron micrographs showing aggregation and ultra-structure characteristics of 14 h-old germlings from the indicated strains. Black arrows, septa; white arrows, cell-to-cell contact area; black star, extracellular matrix.

Plant lectins are used extensively in purification, detection and structural characterization of glyco-conjugates, investigation of cell-surface architecture, blood typing and fractionation of cells [69,70]. Especially plant and invertebrate lectins have proved to be valuable for the detection of specific carbohydrate sequences [71]. Lectin GS II from *Griffonia simplicifolia*, a tetrameric protein with an aggregate molecular weight of ~113 kDa with each site binding a single carbohydrate, is the only known lectin that binds with high selectivity to terminal non-reducing α- and β-*N*-acetyl-D-glucosamine residues of glycoproteins [56–58]. Because of its affinity, lectin GS-II conjugates are useful to identify GlcNAc-containing oligosaccharides. The ability of pre-germinated microconidia (3 h) to bind GS II-FITC conjugate was determined using flow cytometer separation and fluorescence detection. Unexpectedly, in all the experiments the mean values corresponding to the fluorescence emission in Δ*gnt2* mutant cells were significantly higher than those for wild type or the complemented strain (10.5, 7.1, and 4.4 in microconidia, or 12.3, 7.1 and 4.2 in germlings, respectively). To discover the basis for the increased binding capacity, we performed fluorescence analysis using the auxiliary channel adjusted to allow discrimination between single and aggregated cells (Figure 6A). Additionally, the abundance of each cell population was determined for the three strains, by microscopic observation and cell counting. As shown in Figure 6A the percentage of aggregated microconidia in Δ*gnt2* was about 25%, while in the wild type and the complemented strains it was significantly lower (12 and 4%, respectively, *P* < 0.05). Analyses of separate cell populations, single (H3), or aggregated cells (H4) for the three strains are shown in the histograms of relative fluorescence determined by FL1 channel (Figure 6B). The relative fluorescence medium values observed in each segregated cell population were similar for the three strains, around 4 to 6 for single cells and 19 to 25 for aggregated cells. Morphological analysis of cell populations was performed by cytometer light scattering detection, using the forward and side scattered light shape (FSC and SSC, respectively). As represented in Figure 6C, the shapes of the three populations are in accordance to the relative fluorescence value analyses (FL1 channel), showing less uniformity in strains with more aggregated cells. These results were further supported by optical and fluorescence microscopy analysis of GS II-FITC bound cell populations, where we observed a slightly higher number of aggregated cells in the Δ*gnt2* mutant in comparison with the wild type and the complemented strains, resulting in a slightly higher fluorescence intensity of the mutant samples (Figure 7A). 

Recently, it has been reported that changes of the physico-chemical properties of the spore surface may be related to the zeta potential of spores at different pH values [60]. To further analyse the altered aggregation pattern of the Δ*gnt2* mutant, we compared the spore aggregation ability of the different strains in glucose-containing SM at pH values 2.0, 3.5 and 6.0. Conidial aggregation was inhibited at pH 2.0 in all strains, while aggregation was comparable at pH values 3.5 and 6.0. The wild type strain showed around 15-20% aggregation after seven hours incubation at pH 6.0, while this value was up to 40% in the Δ*gnt2* mutant (data not shown).

Comparative TEM analysis of sections through 14 h-old germlings from the wild type strain and the Δ*gnt2* mutant showed abnormally aggregated mutant hyphae, supporting an altered aggregation phenotype in this strain in comparison with the wild type (Figure 7B). Furthermore, Δ*gnt2* hyphae in close proximity appeared to be fixed to each other by a newly existing extracellular matrix of unknown nature, which was not detected in the wild type strain, whose hyphae were not adhered to each other. Detailed analysis of TEM micrographs revealed that Δ*gnt2* hyphae contained aberrant twisted septa compared to those of the wild type, suggesting defects in septum architecture. Despite these findings, no obvious consistent differences in overall cell wall structure were detected.

### *gnt2* mutant cell walls contain reduced levels of N-linked glycans

**Figure 8:**
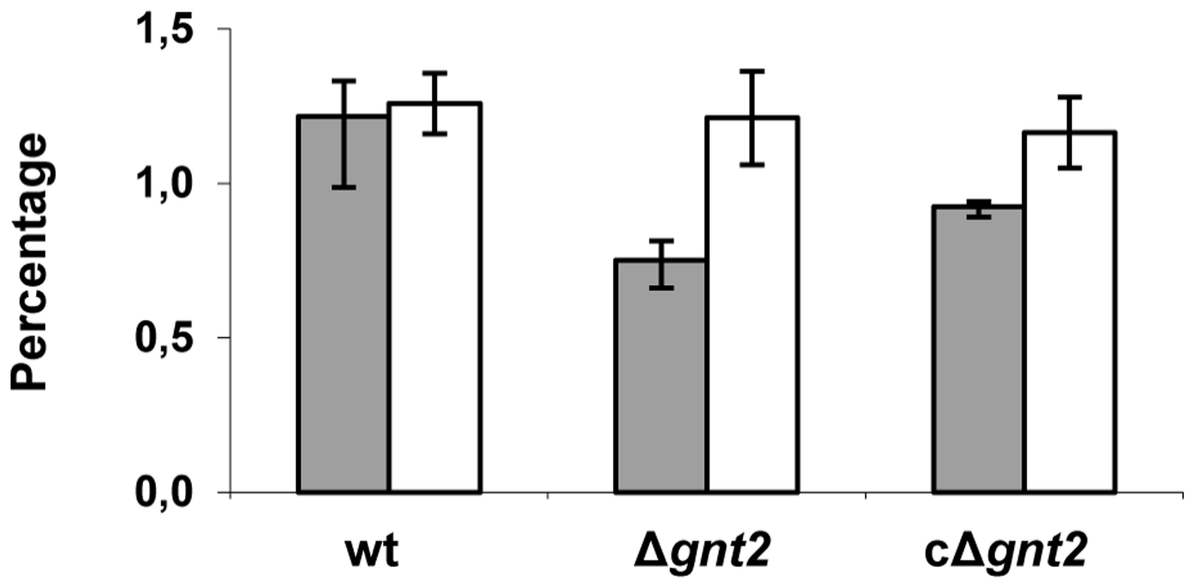
Gnt2 is required for efficient N-glycosylation of cell wall proteins. Protein-associated glycans were released from fungal cell walls in the wild type strain (wt), and the Δ*gnt2* mutant and the cΔ*gnt2* complemented strain by alkali treatment, and the amount of *N*- (grey bars) and *O*-linked glycans (white bars) was calculated relative to cell wall total dry weight. The standard errors from three independent experiments are indicated.

To demonstrate the role of Gnt2 in protein glycosylation, *O*- and *N*-linked glycans were released from cell wall glycoproteins by alkali treatment in the wild type strain, the Δ*gnt2* mutant and the cΔ*gnt2* complemented strain, and estimated relative to total cell wall dry weight. Similar amount of *O*-linked glycans were detected in the three strains, representing 1.2 to 1.25% of the total cell wall biomass (Figure 8). By contrast, the Δ*gnt2* mutant showed a 30% reduction of *N*-linked glycans in comparison with the wild type strain, suggesting a deficient *N*-glycosylation pattern in the mutant strain. The complemented strain partially restored the wild type phenotype.

### GFP::Gnt2 fusion protein localizes in Golgi-like compartments

**Figure 9:**
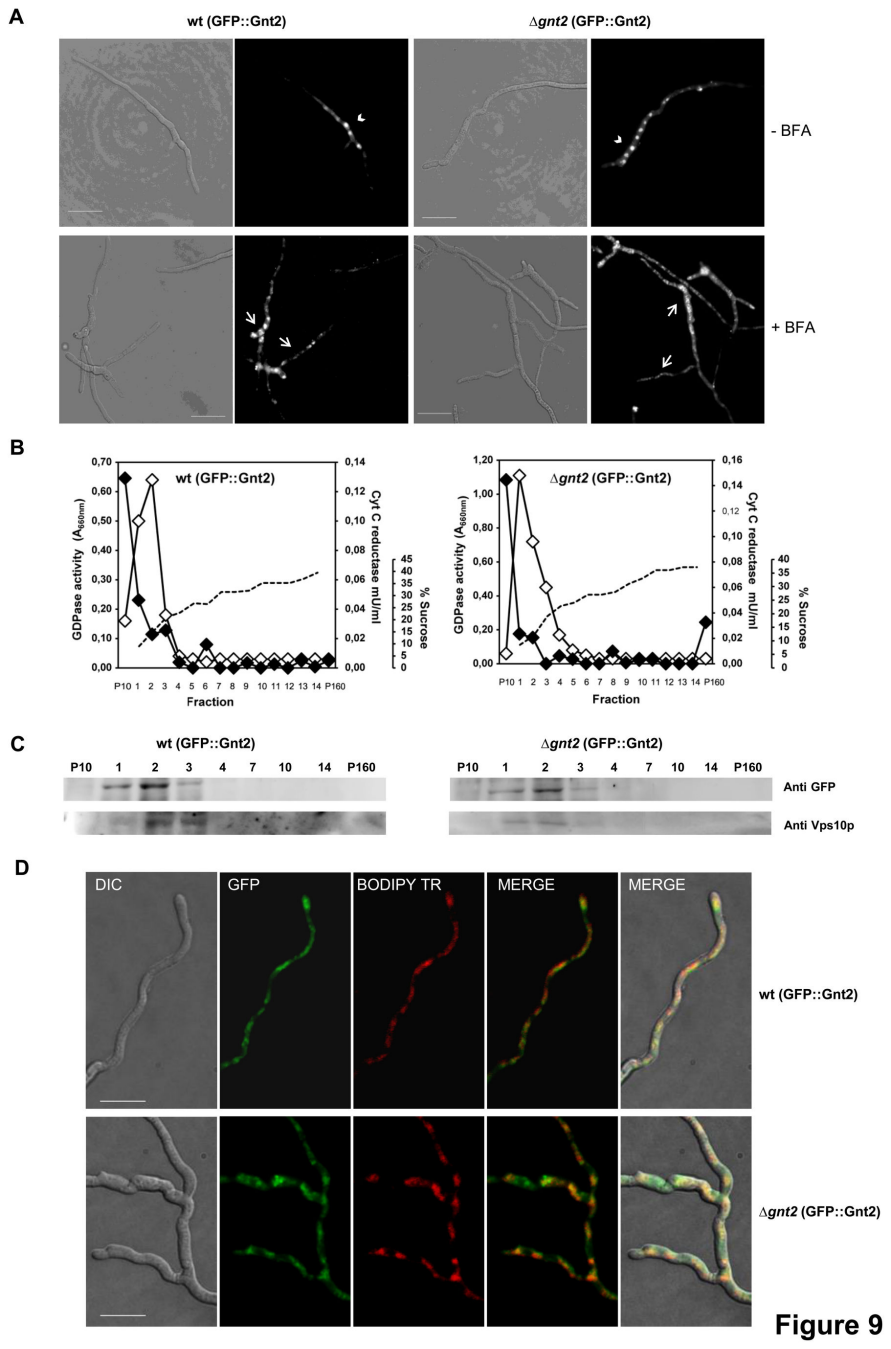
Gnt2 co-localizes with Golgi sub-cellular compartment proteins. (**A**) Light (left panels) and fluorescence (right panels) micrographs of germlings from the wild type (wt) and the Δ*gnt2* mutant, both harbouring the GFP::Gnt2 fusion protein, after 5 min treatment with (+) or without (-) Brefeldin A (BFA). Scale bars, 10 µm. (**B**) Enzymatic activities of sub-cellular fractions (1 to 14) obtained after velocity sucrose gradient ultracentrifugation of cell lysates from the indicated strains. Aliquots of the 10,000 *g* x 10-min pellet (P10) and the 160,000 *g* x 90-min pellet (P160) were also included in the analyses. Dashed line, sucrose concentration; black diamonds, NADPH cytochrome c reductase activity (endoplasmic reticulum marker); white diamonds, GDPase activity (Golgi marker). (**C**) Proteins contained within the indicated fractions were resolved by SDS-PAGE and detected by Western blotting analyses using anti-GFP or anti-Vps10p antibodies, as indicated. (**D**) Colocalization of GFP::Gnt2 (green) with the Golgi apparatus (red) as stained with BODIPY TR ceramide in the indicated strains. Bar, 10 μm.

Gnt2 comprises an N terminal trans-membrane domain and a Golgi retention signal, suggesting that it may be localized to Golgi-like compartments. To investigate its subcellular localization we fused the *GFP* gene encoding the green fluorescent protein in frame to the 5’-end of the *gnt2* gene (Figure S3). This construct was used to transform protoplasts of both the wild type strain and the Δ*gnt2* mutant, resulting in wt (GFP::Gnt2) and Δ*gnt2* (GFP::Gnt2) strains, respectively. Microscopic analysis revealed a strong GFP fluorescence that was enriched in distinct intracellular compartments of both strains (Figure 9A). These large spot-like structures disaggregated into smaller fluorescent spots after treatment with Brefeldin A, which is known to disrupt the Golgi apparatus [72].

To further confirm its localization, mixed vesicle populations from both, the wt (GFP::Gnt2) and Δ*gnt2* (GFP::Gnt2) strains were subjected to centrifugation in a sucrose gradient. Enzymatic activities of specific Golgi and ER markers, GDPase [48] and NADPH cytochrome c reductase, respectively, allowed determination of the enriched Golgi fractions, 1, 2 and 3, as well as the ER enriched fraction P10, in both strains (Figure 9B). Western analyses of those fractions using the specific antibody against the trans-Golgi resident membrane protein Vps10 from *S. cerevisiae* [85] or the anti-GFP antibody confirmed the co-localization of both proteins in the Golgi-enriched fractions (Figure 9C). Localization of Gnt2 was further confirmed by selective staining of Golgi compartments using the red fluorescent BODIPY-TR ceramide in both wt (GFP::Gnt2) and Δ*gnt2* (GFP::Gnt2) strains (Figure 9D). All together these results support the hypothesis that Gnt2 is retained in Golgi-like structures of *F. oxysporum*.

## Discussion

During most host-pathogen interactions, a complex crosstalk between the pathogen and its host is established. In the case of fungal pathogens many infection processes involve two important features: secretion of fungal effector proteins, which interact with a variety of plant responses, and recognition of fungal cell wall proteins during host invasion, which modulates the plant immune response [73–75]. Both fungal secreted effectors and cell wall proteins are generally subjected to post-translational modifications in the endoplasmic reticulum and Golgi apparatus. Protein glycosylation is the most common post-translational modification in eukaryotes and confers the appropriate stability, functionality and localization of cellular proteins [9,10]. Therefore, glycosylation is likely to have an important role in modulating the interaction between *F. oxysporum* and its hosts.

In this study we performed an *in silico* analysis of the *N*- and *O*-glycosylation pathway components from the tomato pathogen *F. oxysporum* f.sp. lycopersici. As reported for the plant pathogen *U. maydis* [34], glycosylation in *F. oxysporum* appears to be simpler, involving less components [24] than the large families described for *S. cerevisiae* or *C. albicans* [76]. Nevertheless, it was remarkable to find seven members of putative *N-*acetyl glucosaminyl transferases (Gnts) in the genome of *F. oxysporum* f.sp. *lycopersici*. This feature appears to be unique for a fungal species, as demonstrated by comparative genomic analysis with the two closely related species *F. graminearum* and *F. verticilloides*, and other related species. This putative glycosyltransferase ‘family’ may have arisen through exon shuffling, or by gene duplication and subsequent divergence. The different number and chromosome location of Gnt encoding genes in *F. oxysporum* in comparison with the two other *Fusarium* *spp.* may point to divergent evolutionary mechanisms during adaptation of the infection process in root and aerial plant pathogens, due to the specific requirements encountered during host colonization [64]. Similar results have been previously reported for cutinolytic encoding genes, in *F. oxysporum* with one single cutinase gene [77], as opposed to three in *F. solani* [78]. 

Gnts are Golgi-localized membrane-bound enzymes involved in key steps of biosynthesis of complex and hybrid *N*-glycans from oligomannose-type *N*-glycans [79]. *Gnt* genes were cloned from various eukaryotic species including mammals, insects, nematodes [80] and higher plants [81,82]. All Gnt proteins have N-terminal transmembrane and conserved central catalytic domains. Since most Gnts are resident and anchored in the membranes lining the ER and the trans-Golgi network (*cis-, medial- trans*-), they contain sequences, in or around their trans-membrane region, for targeting or retention in the Golgi apparatus (mostly unknown) analogous to the K(H)DEL for ER location signal. The *N-*glycans from glycoproteins that move to and through the Golgi may be processed into complex type *N-*glycans by trimming of mannose residues and/or addition of specific sugar residues, before they get to other subcellular organelles, the plasma membrane, the cell wall, or the extracellular space. *Fusarium* Gnts share conserved structural motifs including a transmembrane domain, a Golgi targeting or retention signal, C residues for establishing di-sulfide linkages, and the so-called DXD motifs thought to play a role in metal ion binding and catalysis, and/or DSD as their catalytic active sites for sugar-donor/receptor molecules [81]. We have localized a GFP::Gnt2 fusion protein in intracellular compartments that are sensitive to Brefeldin A and accumulate the fluorescent sphingolipid BODIPY-TR, strongly suggesting that they correspond to *Fusarium* Golgi-like structures. Localization of the fusion protein was further verified by immune-detection in subcellular compartments enriched in GDPase enzymatic activity, described as a Golgi located enzyme in *C. albicans*, *S.* cerevisiae and *Kluyveromyces lactis* [48,83,84], which also contained Vps10, a membrane protein that resides in late-Golgi compartments [85]. 

In order to address the role of *N*-glycosylation in pathogenicity of *F. oxysporum* f.sp. *lycopersici*, we constructed a disruption mutant lacking functional Gnt2. The resulting Δ*gnt2* strain showed a dramatic reduction of its infection capacity on tomato plants, suggesting that correct protein glycosylation may be influencing virulence in this pathogenic fungus. The increased defence-reaction detected in tomato plants inoculated with the Δ*gnt2* mutant might be reflecting recognition of the altered glycosylation pattern of the fungal cell wall surface and/or secreted effectors. Additionally, the Δ*gnt2* mutant showed attenuated virulence on *G. mellonella*, an invertebrate model host that is widely used for the study of microbial human pathogens and has been described as a useful infection model for studying virulence mechanisms of *F. oxysporum* on animal hosts [62]. Protein glycosylation has been identified as an important process for the virulence of various fungal pathogens of animals. To date, most reports have described the importance of *O*-linked glycosylation, frequently *O-*mannosylation. For example, the role of PMTs has been well established to support both morphological transitions and animal host infection by *C. neoformans* [29], *C. albicans* [23–25] and *A. fumigatus* [31]. Other studies have also revealed a role of *N*-glycosylation in virulence of *C. albicans* through the analysis of mutants affected in the subsequent processing of high mannose-type glycans, following the initial transfer of the core oligosaccharide structure onto proteins [26,27]. For plant infecting fungi, to date there are only three examples where defects in protein glycosylation had an effect upon pathogen development and virulence. In the basidiomycete fungus *U. maydis*, mutants lacking an ER associated glycan processing enzyme Glucosidase II grew normally *in vitro* but failed to effectively establish a host–pathogen interface with the plant [35], and the *O*-mannosyltransferase PMT4 was found to be essential for appressorium formation and penetration [32]. Recently, in the wheat leaf infecting fungus *M. graminicola*, the α-1,2-mannosyltransferase MgAlg2, which functions in the early stages of asparagine-linked protein *N*-glycosylation, was shown to play an important role in the virulence of this fungus. Loss of MgAlg2 function gave rise to an inability of spores to extend hyphal filaments, which normally permit leaf penetration by the fungus through stomata [36]. Recently it was found that *N*-glycosylation might also play a role in plant pathogen interactions through functional pattern recognition receptors [86]. In mammalian cells the glycans on the glycoproteins have been proven to be involved in a wide range of biological functions such as receptor binding, cell signalling, protein folding, subcellular distribution and localization, protein stability, endocytosis, immune recognition, inflammation and pathogenicity [9]. Our results on *F. oxysporum* putative *N*-acetylglucosamine transferase Gnt2 suggest that *N*-glycosylation might represent a key virulence mechanism important for the interaction of the pathogen with both plant and animal hosts.

Phenotypic characterization of the Δ*gnt2* mutant indicated hypersensitivity to membrane and cell wall destabilizing agents, as well as to heat stress, and reduced binding affinity to the positively charged dye Alcian Blue. Additionally, septa in the mutant strain showed altered structure when compared with the wild type strain. Despite the fact that no detectable differences in cell wall structures were observed between the wild type and the Δ*gnt2* mutant by TEM, these data suggest that the cell wall and septa in the mutant strain lack the structural strength and/or the physical-chemical properties of wild type cells. This is supported by the observation that mutant cells show a high tendency to aggregate to each other, which could be mediated by the secretion of an extracellular matrix of unknown nature. On the other hand, quantification of cell wall protein-associated glycans revealed a hypo-glycosylated pattern in the Δ*gnt2* mutant, due to either a significant reduction or a different structure of the *N*-linked oligosaccharides, suggesting that Gnt2 is required for efficient *N*-glycosylation of cell wall proteins in *F. oxysporum*. Studies on protein *N*-glycosylation mutants in *S. cerevisiae* pointed that defects in cell wall strength might arise from alterations in the chemical composition of the glycans present in this structure. One hypothesis explaining this link was that under-glycosylated proteins led to incorrectly processed and localized proteins, with loss of enzymatic activities that were critical for biosynthesis of cell wall β1,6-glucan in the mutant strain [87]. A similar explanation was proposed for the *M. graminicola N*-glycosylation mutant Δ*MgAld2*. In this pathogenic fungus *N*-glycosylation plays key roles in regulating the switch to hyphal growth, possibly as a consequence of maintaining correct folding and localization of key proteins involved in this process [36]. The biochemical GlcNAc transferase activity on fungal cell wall proteins has been reported in *C. cinerea*, where depletion of *CcGnt1* gene expression by RNAi strongly reduced the GlcNAc modification in *N*-linked glycans of the silenced transformants fruiting bodies [16].

In conclusion, our results support that *F. oxysporum* f.sp. *lycopersici* contains a putative *N*-Acetyl-glucosaminyl transferase located in Golgi-like compartments essential for virulence and cell wall strength. The precise mechanism underlying these phenotypes is still unknown, but it provides a useful base to analyse in more detail the role of *N*-glycosylation in host-pathogen interactions. A complete comparative analysis of the structure and biosynthesis of cell wall glycans in the Δ*gnt2* mutant will help to understand the immunological properties of the cell wall and the relation between *N*-glycosylation and pathogenesis of *F. oxysporum*.

## Supporting Information

Figure S1 **Disruption of the predicted Gnt2 encoding gene (gnt2).** (**A**) Targeted replacement strategy using the DelsGate technique and the hygromycin resistance cassette (Hyg^R^) as selective marker. Black arrowheads indicate the primer pairs used for amplification of DNA fragments. (**B**) Southern analysis of gDNAs from *F. oxysporum* wild type strain (wt), targeted Δ*gnt2* mutant 55, ectopic transformant #45 and a cΔ*gnt2* mutant complemented with a *gnt2* wild type allele (#6). DNAs were digested with EcoR I and *Xho* I and hybridized with the probe indicated in A (dashed bar).
(TIF)

Figure S2 **Alignment of deduced amino acids from 25 N-acetylglucosamine transferases belonging to eleven fungal species.** *F. oxysporum* (FOXG_01495; FOXG_12436/FOXG_14101; FOXG_12874; FOXG_12897; FOXG_14149; FOXG_16408)*, F. graminearum* (FGSG_10936; FGSG_10956*)*, F. verticillioides* (FVEG_07859; FVEG_11624; FVEG_11649*), *Aspergillus fumigatus* (AFUB_08390), *A. niger* (An11g10206; An03g02990), *A. oryzae* (AO09001000), *Chaetomium globosum* (CHGG_09837; CHGG_08452), *Saccharomyces cerevisiae* (GNT1, YOR320C), *Nectria haematococca* (NHA_39806; NHA_79931; NHA_79932), *Verticillium dahliae* (VDBG_01023; VDBG_03984), *Penicillium chrysogenum* (PC_16g00820; PC_22g12650). The alignment corresponds to the G-block used for construction of the cladogram in Figure 2.
*These genes were manually cured as follows: FGSG_10956, 120 amino acids were added upstream of the annotated start codon; FVEG_11649, 78 amino acids were added upstream of the annotated start codon.
(EPS)

Figure S3 **Construction of the GFP::Gnt2 fusion protein.** The *gnt2* cDNA fragment was amplified with the indicated primers, containing the *Nsi* I and *Xma* I restriction sites inserted at 5´or 3´ end of the ORF, respectively, and cloned into the corresponding restriction sites of the expression vector p1902. The resulting in-frame ORF fusion is under control of the *Aspergillus nidulans* *gpdA* promoter and terminator. Arrows indicate the transcriptional orientation.
(TIF)

Table S1 **Identification of *Fusarium oxysporum* orthologue genes** involved in glycosylation pathways by comparison to those described in *Saccharomyces cerevisiae*. For protein blast search in the Broad Institute database http://www.broadinstitute.org/annotation/genome/fusarium_group/.
the S. *cerevisiae* proteins were used as query sequences. The systematic name for *S. cerevisiae* genes and their function were obtained from *Saccharomyces* genome database (http://www.yeastgenome.org/). *F. oxysporum* gene numbers and *E* value parameter after WUBLAST analysis were obtained directly from http://www.broadinstitute.org/annotation/genome/fusarium_group/.
(DOCX)
