# Supplementary figures and images for: The Fusarium oxysporum gnt2, Encoding a Putative N-Acetylglucosamine Transferase, Is Involved in Cell Wall Architecture and Virulence
Source: PLoS One. 2013 Dec 27;8(12):e84690. doi: 10.1371/journal.pone.0084690 (PMC3886883; doi:10.1371/journal.pone.0084690)

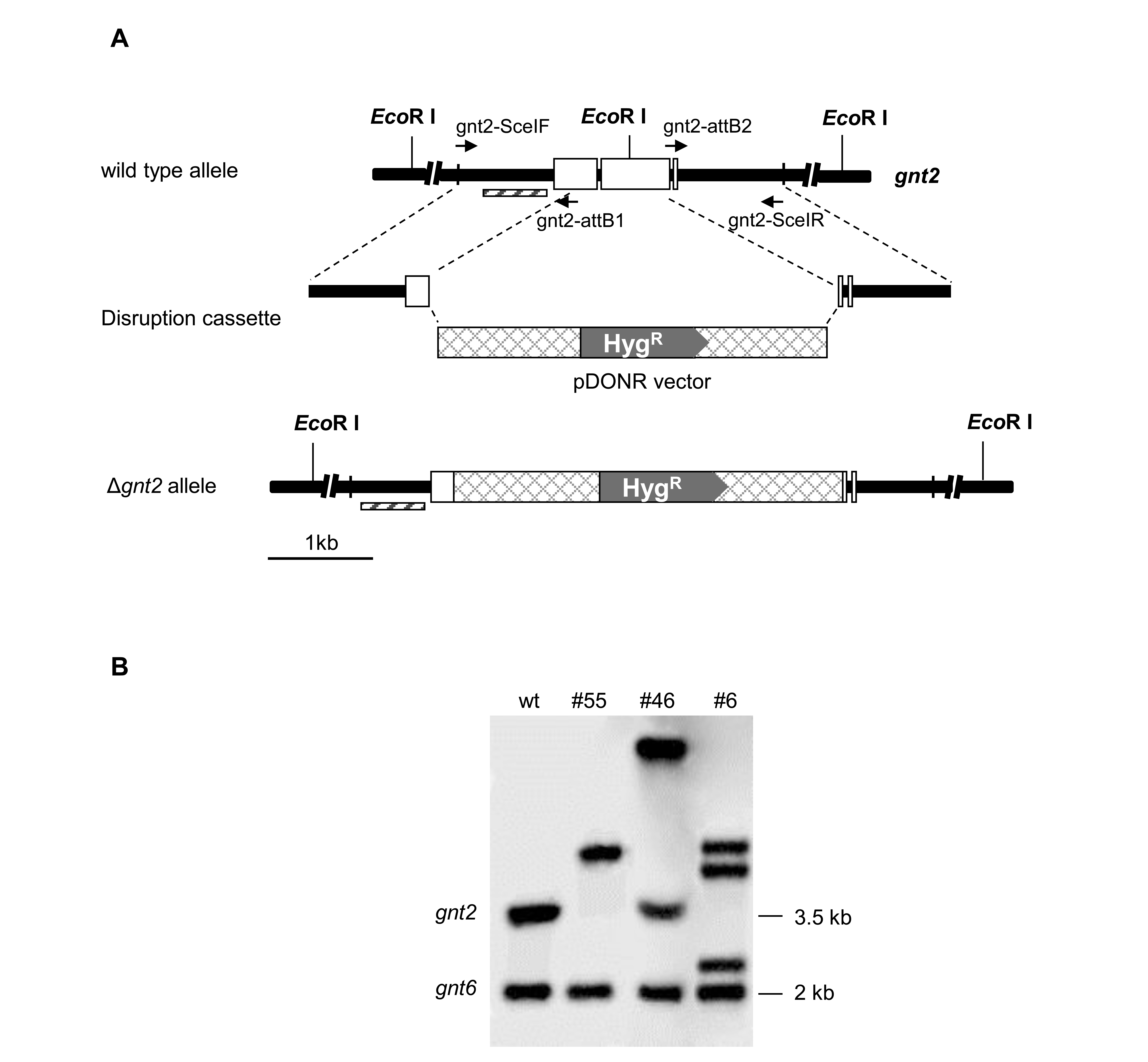

Supplement: Figure S1 — Disruption of the predicted Gnt2 encoding gene (gnt2). (A) Targeted replacement strategy using the DelsGate technique and the hygromycin resistance cassette (HygR) as selective marker. Black arrowheads indicate the primer pairs used for amplification of DNA fragments. (B) Southern analysis of gDNAs from F. oxysporum wild type strain (wt), targeted Δgnt2 mutant 55, ectopic transformant #45 and a cΔgnt2 mutant complemented with a gnt2 wild type allele (#6). DNAs were digested with EcoR I and Xho I and hybridized with the probe indicated in A (dashed bar). (TIF) [file pone.0084690.s001.tif]
