# Supplementary material for: The Fusarium oxysporum gnt2, Encoding a Putative N-Acetylglucosamine Transferase, Is Involved in Cell Wall Architecture and Virulence
Source: PLoS One. 2013 Dec 27;8(12):e84690. doi: 10.1371/journal.pone.0084690 (PMC3886883; doi:10.1371/journal.pone.0084690)
Supplement: Table S1 — Identification of Fusarium oxysporum orthologue genes involved in glycosylation pathways by comparison to those described in Saccharomyces cerevisiae. For protein blast search in the Broad Institute database http://www.broadinstitute.org/annotation/genome/fusarium_group/. the S. cerevisiae proteins were used as query sequences. The systematic name for S. cerevisiae genes and their function were obtained from Saccharomyces genome database (http://www.yeastgenome.org/). F. oxysporum gene numbers and E value parameter after WUBLAST analysis were obtained directly from http://www.broadinstitute.org/annotation/genome/fusarium_group/. (DOCX) [file pone.0084690.s004.docx]

**Table S1 Identification of *Fusarium oxysporum* orthologue genes** involved in glycosylation pathways by comparison to those described in *Saccharomyces cerevisiae*. For protein blast search in the Broad Institute database http://www.broadinstitute.org/annotation/genome/fusarium_group/

the *S. cerevisiae* proteins were used as query sequences. The systematic name for *S. cerevisiae* genes and their function were obtained from *Saccharomyces* genome database (http://www.yeastgenome.org/). *F. oxysporum* gene numbers and *E* value parameter after WUBLAST analysis were obtained directly from http://www.broadinstitute.org/annotation/genome/fusarium_group/.

| *S. cerevisiae* | | |  | *F. oxysporum* | | |
| --- | --- | --- | --- | --- | --- | --- |
| Protein | Gene | Function |  | Gene | E-value | SL |
| Alg1 | YBR110W | β-1,4 mannosyltransferase (1^st^ Mannose) in *N-*glycosylation |  | FOXG_06338 | 0 | ER |
| Alg2 | YGL065C | α-1,3 mannosyltransferase (2^nd^ Mannose) in *N-*glycosylation |  | FOXG_11063 | 0 | ER |
| Alg3 | YBL082C | α-1,3 mannosyltransferase (6^th^Mannose) in *N-*glycosylation |  | FOXG_08415 | 1,4 e^-42^ | ER |
| Alg4 | YFL045C | Phospho-mannomutase, synthesis of GDP-mannose and Dol-P N*-*glycosylation |  | FOXG_01989  FOXG_16804 | 0  1,4 e^-9^ | C |
| Alg5 | YPL227C | Dolichyl-Phosphate-glucosyltransferase involved in *N-*glycosylation |  | FOXG_08496 | 0 | ER |
| Alg6 | YOR002W | α-1,3- glucosyltransferase  dolicholglucosyltransferase (1^st^ Glucose)in *N-*glycosylation |  | FOXG_09280 | 0 | ER |
| Alg7 | YBR243C | UDP-N-acetyl-Glucosamine-1P-Transferase (1^st^ GlcNAc) involved in *N-*glycosylation |  | FOXG_06286 | 0 | ER |
| Alg8 | YOR067C | α-1,3 glucosyltransferase (2^nd^ Glucose) in *N*-glycosylation |  | FOXG_10557 | 0 | ER |
| Alg9 | YNL219C | α-1,2 mannosyltransferase (7^th^ and 9^th^ Mannoses) in *N*-glycosylation |  | FOXG_08463 | 1,18 e^-30^ | ER |
| Alg10 | YGR227W | α-1,2 glucosyltransferase (3^rd^ Glucose) in *N-*glycosylation |  | FOXG_08401 | 5,1 e^-17^ | ER |
| Alg11 | YNL048W | α-1,2 mannosyltransferase (4^th^ and 5^th^ Mannoses) in *N*-glycosylation |  | FOXG_12797 | 0 | ER |
| Alg12 | YNR030W | α-1,6 mannosyltransferase (8^th^ Mannose) in *N*-glycosylation |  | FOXG_00155 | 2,77 e^-33^ | ER |
| Cne1 | YAL058W | Calnexin (chaperone for quality control and correct folding of glycoproteins) |  | FOXG_00565 | 0 | ER |
| Dpm1 | YPR183W | Dolichol phosphate mannose synthase involved in *O*-and *N*-glycosylation |  | FOXG_11608 | 1,7 e^-22^ | ER |
| Gls1/ Cwh41 | YGL027C | α-glucosidase I, removes 3^rd^ glucose, from Man_9_Glc_3_GlcNAc_2_ in N-glycosylation |  | FOXG_07965 | 0 | ER |
| Gls2/ Rot2 | YBR229C | α-glucosidase II catalytic subunit, removes 2^nd^ and 1^st^ glucoses from Man_9_Glc_2_GlcNAc_2_ in N-glycosylation |  | FOXG_07678 | 0 | ER |
| Gna1 | YFL017C | glucosamine 6-P acetyltransferase, synthesis of UDP-GlcNAc |  | FOXG_03678  FOXG_03874 | 4,7 e^-24^  7,7 e^-22^ | C |
| Gnt1 | YOR320 | *N-*acetylglucosaminetransferase, modifies *N-*glycoproteins |  | FOXG_12874  FOXG_12897  FOXG_01495  FOXG_14149  FOXG_12436  FOXG_14101  FOXG_16408 | 9,6 e^-23^  2,6 e^-22^  9,7 e^-20^  3,7 e^-13^  1,9 e^-12^  1,9 e^-12^  3,4 e^-8^ | G |
| Hoc1 | YJR075W | α-1,6 mannosyltransferase involved in cell wall mannan biogenesis in *N*- glycosylation |  | FOXG_06111  FOXG_03626  FOXG_10373 | 0  3 e^-30^  3 e^-28^ | G |
| Ire | YHR079C | Serin-threonin kinase and endoribonuclease |  | FOXG_08844 | 0 | ER |
| Kar2 | YJL034W | ATPase involved in protein import into the ER |  | FOXG_06136 | 0 | ER |
| Kre2/ Mnt1 | YDR483W | α-1,2 mannosiltransferase involved in *O-* and *N-*glycosylation |  | − |  | G |
| Kre5 | YOR336W | UDP glucose:glicoprotein glycosyltransferase in *N*-glycosylation |  | FOXG_03728 | 1,37 e^-19^ | ER |
| Kre6 | YPR159W | Glucosidase |  | − |  | ER |
| Ktr1 | YOR099W | α-1,2 mannosyltransferase involved in *O*- and *N-*glycosylation |  | FOXG_08407  FOXG_06418 | 0  5,06 e^-37^ | G |
| Ktr2 | YKR061W | mannosyltransferase involved in *N*-glycosylation |  | − |  | G |
| Ktr3 | YBR205W | α-1,2 mannosyltransferase involved in *O*- and *N*-glycosylation |  | − |  | G |
| Ktr4 | YBR199W | Putative mannosyltransferase |  | FOXG_01521 |  | G |
| Ktr5 | YNL029C | Mannosyltransferase involved in *N*-glycosylation |  | FOXG_10333 | 0 | G |
| Ktr6/ Mnn6 | YPL053C | Mannosyltransferase involved in sugar core formation in *N*-glycans |  | − |  | G |
| Ktr7 | YIL085C | Mannosyltransferase involved in *O*- and *N*-glycosylation |  | − |  | G |
| Mnl1 | YHR204W | Exomannosidase like protein, in complex with PDI1 removes 8^th^ mannose and produces Man_7_GlcNAc for proteasome degradation |  | FOXG_05493 | 4,9 e^-23^ | ER |
| Mnn1 | YER001W | α-1,3 mannosyltransferase involved in *O-* and *N*-glycosylation |  | FOXG_05718  FOXG_12231 | 1,3 e^-15^  1,3 e^-9^ | G |
| Mnn2 | YBR015C | α-1,2 Golgi mannosyltransferase, adds 1^st^ mannose to the branches |  | FOXG_12373 | 2,6 2^-39^ | G |
| Mnn4 | YKL201C | Putative positive regulator of mannosylphosphate transferase (Ktr6p) in *O*- and *N*-glycosylation |  | − |  | G |
| Mnn5 | YJL186W | α-1,2 mannosyltransferase from early Golgi, adds 2^nd^ mannose to sugar branches |  | − |  | G |
| Mnn8/ Anp1 | YEL036C | Subunitfrom α-1,6 mannosyltransferase complex involved in *N-*glycosylation |  | FOXG_11346  FOXG_05317 | 0 | G |
| Mnn9 | YPL050C | Subunit α-1,6 of mannosyltransferase complex involved in *N*-glycosylation |  | FOXG_01971 | 0 | G |
| Mnn10 | YDR245W | Subunit α-1,6 mannosyltransferase complex involved in *N*-glycosylation |  | FOXG_01933 | 0 | G |
| Mnn11 | YJL183W | Subunit α-1,6 mannosyltransferase complex involved in *N*-glycosylation |  | FOXG_13822 | 1,4 e^-22^ | G |
| Mns1 | YJR131W | α-1,6 mannosydase removing 9^th^ mannose producing Man_8_GlcNAc_2_ in *N*-glycosylation |  | FOXG_00435 | 0 | ER |
| Mnt3 | YIL014W | α-1,3 mannosyltransferase, adds 4^th^ & 5^th^ mannoses to *O*-linked glycan |  | − |  | G |
| Mnt4 | YNR059W | α-1,3 Putative α-1,3 mannosyltransferase, dispensable for *O*-glycosylation |  | − |  | G |
| Och1 | YGL038C | Mannosyltransferase enlarge *N*-linked-glycans adding the first α-1,6 mannose in early Golgi |  | FOXG_06111 | 0 | G |
| Ost1 | YJL002C | α subunit of oligosaccharyltransferase complex (OST) involved in *N*-glycosylation |  | FOXG_00557 | 5,6 e^-45^ | ER |
| Ost2 | YOR103C | Subunit ε from oligosaccharyltransferase complex (OST) involved in *N*-glycosylation |  | FOXG_01713 | 1,9 e^-18^ | ER |
| Ost3 | YOR085W | Subunit γ from oligosaccharyltransferase complex (OST) involves in *N*-glycosylation |  | FOXG_05558 | 8,7 e^-18^ | ER |
| Ost4 | YDL232W | Subunit from oligosaccharyltransferase complex (OST) involves in *N*-glycosylation |  | − |  | ER |
| Ost5 | YGL226C-A | Subunit z from oligosaccharyltransferase complex (OST) involves in *N*-glycosylation |  | − |  | ER |
| Ost6 | YML019W | Subunit from oligosaccharyltransferase complex (OST) involves in *N*-glycosylation |  | − |  | ER |
| Pcm1 | YEL058W | *N*-acetyl glucosamine-phosphate mutase, from GlcNAc-6P to GlcNAc-1-P, chitin biosynthesis, *N*-glycoproteins and GPI-anchored |  | FOXG_00256 | 0 | C |
| Pdi1 | YCL043C | Protein disulfide isomerase, multifunctional ER protein, essential for disulfide bridges in surface and secreted proteins, in complex with MNL1, removes 8^th^ mannose and produces Man7GlcNAc for proteosome degradation |  | FOXG_00140 | 0 | ER |
| Pmr1/ SSC1 | YGL167C | ATPase Ca^2+^dependent |  | − |  | G |
| Pmt1 | YDL095W | *O*-mannosyltransferase |  | FOXG_03668 | 0 | ER |
| Pmt3 | YOR321W | *O*-mannosyltransferase |  | − |  | ER |
| Pmt4 | YJR143C | *O*-mannosyltransferase |  | FOXG_00440 |  | G |
| Pmt5 | YDL093W | *O*-mannosyltransferase |  | − |  | G |
| Pmt6 | YGR199W | *O*-mannosyltransferase |  | − |  | G |
| Png1 | YPL096W | *N*-glycanase, required for de-glycosylation of miss-formed protein |  | FOXG_01189 | 0 | C |
| Qri1 | YDL103C | UDP-*N*-acetylglucosaminepyrophosphorylase, *N*-glycoproteins and GPI-anchored |  | FOXG_00832 | 0 | C |
| Rft1 | YBL020W | Flipase, translocation of Man_5_GlcNAc_2_-PP-Dol from ER cytoplamic side to the lumenal ER membrane |  | FOXG_10617 | 0 | ER |
| Sec59 | YMR013C | Dolichol kinase, biosynthesis of Dol-P in *N*-glycosylation |  | FOXG_07836 | 4,9 e^-19^ | ER |
| Stt3 | YGL022W | Subunit of the oligosaccharyltransferase complex (OST) involved in N-glycosylation |  | FOXG_00621 | 0 | ER |
| Van1 | YML115C | α-1,6 mannosyltransferase involved in *N*-glycosilation |  | FOXG_05317 | 0 | G |
| Vrg4/ Van2 | YGL225W | Transporter for GDP-mannose |  | FOXG_10351  FOXG_14501 | 0  0 | G |
| Yur1 | YJL139C | α-1,2 mannosyltransferase involved in *N-*glycosylation (paralog of Ktr2) |  | − |  | G |
| Wbp1 | YEL002C | β subunit of the oligosaccharyltransferase glycoprotein complex (OST) |  | FOXG_01288 | 8,3 e^-44^ | ER |
